# Clients’ experiences utilizing a safer conception service for HIV affected individuals: implications for differentiated care service delivery models

**DOI:** 10.1186/s12978-019-0718-5

**Published:** 2019-05-29

**Authors:** Sheree Schwartz, Natasha Davies, Nicolette Naidoo, Diantha Pillay, Nokuthula Makhoba, Saiqa Mullick

**Affiliations:** 1 Department of Epidemiology, Johns Hopkins School of Public Health, Baltimore, MD USA; 2 Wits Reproductive Health Institute, University of the Witwatersrand, Johannesburg, South Africa

**Keywords:** Safer conception, HIV, HIV prevention, Differentiated care, Discordant couples, Implementation

## Abstract

**Background:**

Safer conception services promote the reproductive health and rights of families, while minimizing HIV transmission risks between partners trying to conceive, as well vertical transmission risks. Implementation data, including clients’ experiences utilizing safer conception services in sub-Saharan Africa are limited.

**Methods:**

Hillbrow Community Health Centre began offering safer conception services for individuals and couples affected by HIV in Johannesburg, South Africa in June 2015. A stratified sub-sample of safer conception clients were consecutively recruited from April 2016–August 2017 for a cross-sectional interview assessing clients’ perceptions of service acceptability and value, as well as perceived safer conception knowledge and self-efficacy. Visual analog scales from 0 to 100 were used to measure clients’ experiences; scores were classified as low, moderate and high acceptance/value/knowledge/self-efficacy if they were < 50, 50–79 and ≥ 80 respectively. Comparisons of scores were made across safer conception visits attended.

**Results:**

Among 692 clients utilizing safer conception services, 120 (17%) were sampled for the process evaluation; sub-sample participant characteristics were similar to the overall cohort. Clients gave a mean score of ≥90-points for each question assessing service acceptability and 96% (114/119) indicated a high perceived value (scores ≥80) for regular safer conception attendance until conception. Fifty-eight percent (*n* = 70) of clients reported learning something new during the visit completed the day of the survey, though acquisition of new information tended to decrease as visits increased (*p* = 0.09). In terms of safer conception strategies, 80% of clients reported high levels of knowledge on the impact of antiretroviral treatment (ART) and viral suppression on HIV transmission, 67% reported high levels of knowledge of the importance of STI screening and 56% regarding limiting condomless sex to days of peak fertility; 34% in sero-different relationships reported high pre-exposure prophylaxis (PrEP) knowledge. Self-efficacy varied by safer conception methods and was similar across study visits.

**Conclusions:**

Clients perceived high value from their safer conception visits and preferred regular attendance until conception, however we observed a plateau in knowledge and self-efficacy across subsequent visits after initially attending safer conception care. More intensive services may be appropriate for certain clients based on clinical circumstances, but many couples may potentially receive a ‘lighter touch’ approach while still minimizing HIV transmission risks.

**Electronic supplementary material:**

The online version of this article (10.1186/s12978-019-0718-5) contains supplementary material, which is available to authorized users.

## Background

HIV combination prevention efforts, including expanded access to antiretroviral treatment (ART) for individuals living with HIV, need to be implemented at scale to change the trajectory of the HIV epidemic [1]. To this end, UNAIDS has introduced ambitious 90–90-90 targets and a recent HIV prevention roadmap [2], with the goal of ending the HIV epidemic by 2020 [3]. However, even within the current state of expanded treatment availability in sub-Saharan Africa, there remain populations that have elevated risks for HIV acquisition and transmission [4]. There are few tested, scalable service delivery models providing comprehensive services that cater for specific needs of these at-risk groups, including HIV affected couples trying to conceive [5–7].

Individuals and couples affected by HIV who are trying to conceive, particularly those in HIV sero-different relationships where one partner is HIV-positive and the other HIV-negative, have specific HIV prevention needs that arise from their reproductive goals and should be supported as part of universal access to sexual and reproductive health and rights (SRHR) [8]. Studies have consistently shown that people living with HIV commonly desire to have children, but lack information about how to conceive safely [9–11]. Consequently, HIV affected couples often try to become pregnant prior to optimizing their health, which is important to reduce HIV transmission and acquisition risks [12–14].

Safer conception services provide a comprehensive approach to promote the SRHR of families, while minimizing the HIV transmission risks between partners trying to conceive, as well as the onward vertical transmission risks to children of mothers living with HIV [15]. Combination HIV prevention methods for safer conception in low resource settings may include immediate ART initiation for HIV-positive partners, monitoring of viral loads and confirmed pre-conception viral suppression, screening and treatment of sexually transmitted infections (STIs), education around estimated ovulation dates and peak fertility for timed condomless sex or self-insemination with a needleless syringe, and initiation of pre-exposure prophylaxis (PrEP) for HIV-negative partners [16].

There is increasing recognition that individuals or populations have different HIV prevention and treatment needs, and that differentiated service delivery models may help to achieve ambitious treatment targets by efficiently allocating resources and providing care tailored to patient preferences [17]. Considerations for differentiated care models include where and when services are provided, who is providing the services and what package of care is offered [18]. Safer conception care is itself a differentiated care model, providing a package of services tailored to meet the specific needs of HIV affected couples trying to become pregnant.

Although the calls for safer conception service provision have escalated in recent years, few primary healthcare safer conception services have been introduced in sub-Saharan Africa and implementation data are only beginning to emerge [19–21]. Data on clients’ experiences utilizing safer conception services are to-date unavailable. However, actual client data are critical to assess the acceptability and potential impact of safer conception services, as well as to optimize service delivery models for scale-up. The objective of this study is to evaluate the acceptability, perceived value, knowledge and self-efficacy among HIV affected couples attending safer conception services in Johannesburg, South Africa.

## Methods

### Study setting and population

Safer conception services were initiated within the Hillbrow Community Health Centre ART clinic in June 2015, as a proof of concept demonstration project to assess the feasibility, acceptability and effectiveness of safer conception services. The ART program serves more than 21,000 HIV-positive patients in the densely populated, urban center of Johannesburg. The safer conception service is a standalone service based at the ART clinic, with referrals received from various entry points at the community health center, as well as other nearby clinics. Entry points within the community health center include the ART clinic, family planning, HIV testing and counseling, general primary healthcare services, the male medical circumcision program and mobile van services. Demand generation is created through health talks, posters, referrals from counselors and clinicians at the various entry points and word of mouth.

The safer conception package includes HIV counseling and testing, screening for STIs and cervical cancer, ART initiation and management, viral load monitoring, counseling on the fertile window and timed condomless sex, self-insemination instruction, PrEP and partner HIV disclosure support. PrEP was initially only available through prescriptions to private pharmacies, but became available at the clinic on an individual basis as availability within the country’s public health sector increased. Safer conception services are nurse-driven, with additional supervision and care available from a doctor, and HIV counseling and testing offered through a lay counsellor. Clients were seen every 1–2 months. Initial consultations lasted around 45 min, with follow-up visits typically ranging from 15 to 30 min.

Men and women were eligible to receive services and participate in the research if they were 18 years or older, in a relationship in which at least one partner was HIV-positive, planned to attempt pregnancy within the next six months and had not been given a prior, unresolved infertility diagnosis.

### Study design

The demonstration project uses an observational cohort design, such that clients are educated on safer conception strategies, and in consultation with their safer conception clinical provider, decide which safer conception strategies to use given their circumstances. Services begin with HIV testing and STI screening, but then diverge based on patient preferences, sero-dynamics and partner involvement. Follow-up of the primary safer conception effectiveness endpoints, including pregnancy incidence and HIV transmission, will be subsequently reported.

As part of the larger safer conception demonstration project, we sought to evaluate implementation-related outcomes through a quantitative process evaluation, with the goal of gaining insights into mechanisms of intervention success and challenges, and to inform potential service delivery scale-up. Acceptability of the service delivery approach, perceived value of services and knowledge acquired, as well as self-efficacy to implement safer conception strategies were core elements evaluated.

The evaluation was conducted in a sub-sample of safer conception clients. Clients enrolled in the safer conception study and attending safer conception services between April 2016 and August 2017, were consecutively approached and offered participation in the process evaluation upon completion of their safer conception visit. As the purpose of the evaluation was to assess patient experiences rather than to evaluate efficacy or effectiveness, a sample size of 120 (intended to be 15–20% of the cohort) was targeted. Stratified sampling was used such that at least 25 clients were represented from those completing a first visit, second visit, third visit, or four or more visits to ensure representation across a breadth of experiences among safer conception attendees.

Ethical approval was provided by the Human Research Ethics Committee of the University of the Witwatersrand (protocol *M150146*). All participants completed written informed consent.

### Data collection and analyses

Process evaluation data were collected through a one-time, interviewer-administered survey. The surveys were tablet-based and captured in REDCap, a secure web-based application designed for data capture and management [22]. Surveys were conducted in a private space with confidentiality of responses emphasized. Interviews lasted approximately 15 min and drew heavily from use of visual analog scales (VAS) allowing clients to rate their experience on a scale of 0–100 [23].

Descriptive analyses broadly assessed safer conception service acceptability and perceived value, knowledge and self-efficacy. Acceptability of safer conception services was measured using a VAS assessing patient comfort being seen with a partner, acceptability around total time spent at the service and comfort with the privacy/confidentiality of the service. Acceptability and perceived value were further assessed through a VAS rating of perceived value of regular safer conception service attendance until pregnancy is achieved, preferred frequency of safer conception visits, the proportion learning something new during their visit and a VAS rating of the perceived utility of the information received during the visit. Aspects of disclosure to partners, where applicable, were additional measures of acceptability and value of the service measured through VAS. Perceived knowledge of, and self-efficacy to use, various safer conception methods were self-reported using the VAS rating. Client confidence in the effectiveness of the services to reduce their horizontal and vertical HIV transmission risks was also reported. In addition to reporting means and standard deviations of the VAS scores, participants were classified as perceiving low, moderate and high acceptance/value/knowledge/self-efficacy levels if they reported VAS scores of < 50, 50–79 or ≥ 80 respectively. Clients were then classified according to these threshold levels. Comparisons were made by gender and across study visit category (e.g. first, second, third or four or more visits) using chi-squared statistics to compare categorical variables and t-tests or analysis of variance statistics (ANOVA) to compare mean VAS scores across two or three or more groups respectively. Finally, VAS scores for knowledge and self-efficacy around safer conception methods were ranked within individuals as a sensitivity analysis to compare knowledge and self-efficacy between methods [24]. For example, we assessed what percentage of clients reported that the method for which they had the highest level of safer conception knowledge was viral suppression vs. self-insemination vs. PrEP use, etc. Analyses were performed in Stata 14.1 (College Station, Texas).

## Results

Among the 692 clients taking up safer conception care, 120 (17%) were sampled for the process evaluation, including 78 women and 42 men. To assess the representativeness of the sub-sample, we compared client characteristics of the overall safer conception cohort to characteristics of the process evaluation participants (Table 1). Clients participating in the process evaluation were comparable to the overall safer conception cohort.

**Table 1:** Characteristics of Safer Conception Clients Overall and Clients Completing the Process Evaluation Survey

Characteristics	Clients in overall cohort (*n* = 692)	Clients completing process evaluation (*n* = 120)
Age, median (IQR)	34 [30–37]	33 [31–37]
Gender, n (%)		
Female	454 (66%)	78 (65%)
Male	238 (34%)	42 (35%)
Nationality, n (%)		
South African	379 (55%)	67 (56%)
Non-South African	313 (45%)	53 (44%)
Employment status, n (%)^a^		
Employed	490 (71%)	85 (71%)
Unemployed	200 (29%)	35 (29%)
Education completed, n (%)		
Grade school or less	67 (10%)	13 (11%)
9-12th grade	236 (34%)	38 (32%)
Matriculated from high school	318 (46%)	61 (51%)
Tertiary studies	66 (10%)	8 (6%)
Income, median ZAR [IQR]^b^	3300 [1500–5000]	3300 [1600–5150]
HIV Status, n (%)		
HIV-positive	591 (86%)	109 (91%)
HIV-negative	99 (14%)	11 (9%)
On ART at baseline if HIV-positive, n (%)		
Yes	532 (90%)	100 (92%)
No	60 (10%)	9 (8%)
Relationship sero-dynamics		
Sero-different	143 (30%)	39 (33%)
HIV+ sero-same	219 (46%)	69 (57%)
HIV+ client with partner of unknown status	117 (24%)	12 (10%)

^a^Data missing for *n* = 2. ^b^Data missing for *n* = 19.

Safer conception clients participating in the process evaluation had completed a median of three [interquartile range (IQR) 2–4] safer conception visits at the time of survey administration. Twenty-one percent (*n* = 25) completed the interview after their first safer conception visit, 27% (*n* = 32) after their second visit, 22% (*n* = 27) after their third visit and 30% (*n* = 36) after attending four or more visits.

### Acceptability and perceived value of safer conception services

**Fig. 1:**
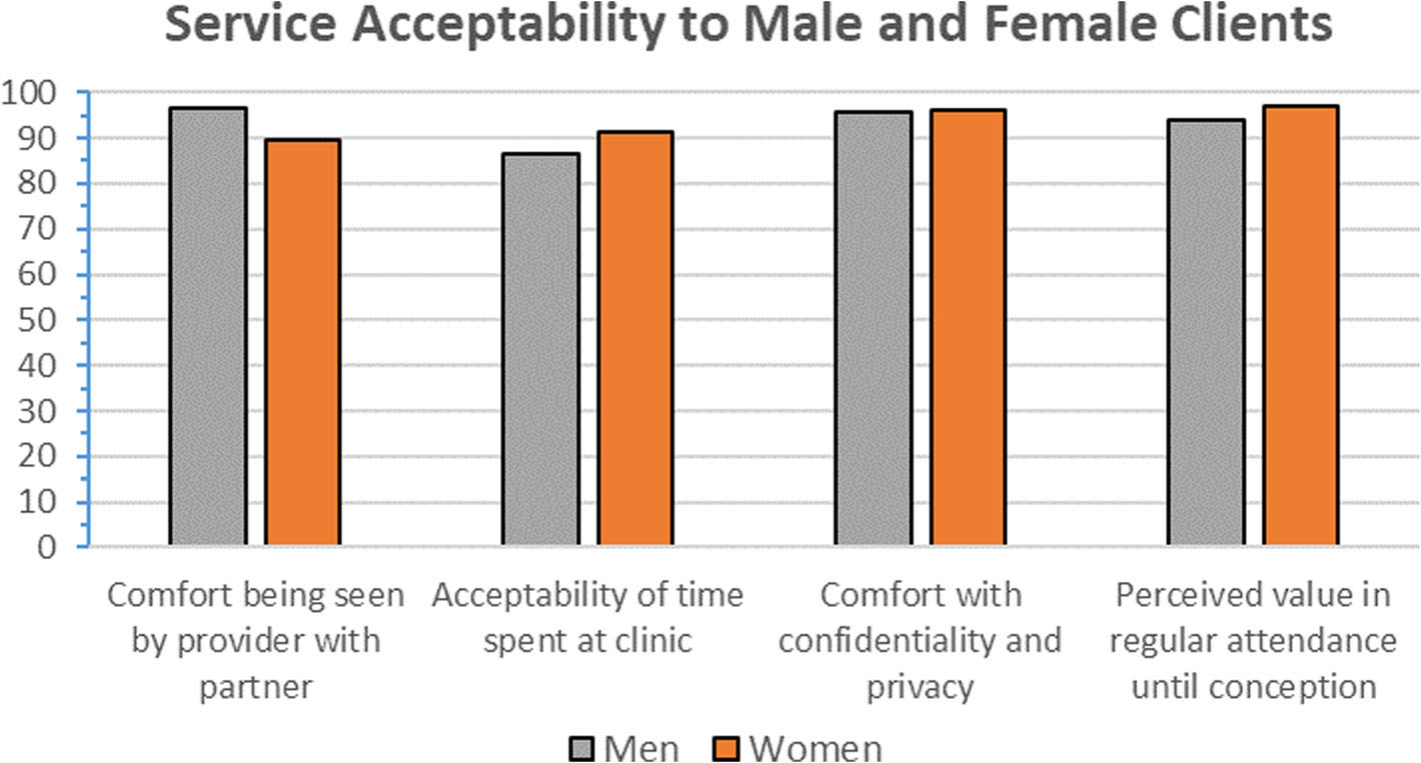
Service acceptability among men and women attending safer conception care in Johannesburg, South Africa (2016–2017)

Acceptability of the service was high. Participants gave a mean score of 90-points or higher for each question assessing acceptability, including comfort in being seen by their healthcare provider in the presence of their partner, time spent at the clinic, and the level of confidentiality and privacy offered (Fig. 1). Findings did not differ between men and women. Overall, 57% (*n* = 68/120) of clients completing the process evaluation had attended at least one safer conception visit with their partner at the time of the interview.

Among those who had not attended with a partner, 62% (*n* = 32/52) indicated that their partner intended to accompany them on a future visit. Eight clients had not disclosed their HIV status to their partner at the time of the process evaluation. In terms of acceptability and value specific to the disclosure intervention (*n* = 8), 63% perceived the advice received around disclosure for safer conception purposes to be of a high value (25% moderate, 12% low). Fifty percent reported low comfort levels discussing disclosure with providers (25% moderate, 25% high), though perceived pressure from healthcare providers to disclose to their partner was generally low (75% reported low levels, 25% high).

Overall, 96% (*n* = 114/119) indicated a high perceived value for the importance of regular attendance of the safer conception clinic until conception was achieved (VAS score of ≥80). The perceived importance of regular attendance was similar by gender (Fig. 1), as well as by the number of safer conception visits the client had already completed (results not shown). Similarly, when asked about preferred visit attendance frequency, 41% (*n* = 49) of clients preferred monthly visits, 21% (*n* = 25) preferred every two months, 32% (*n* = 38) every three months, 2% (n = 3) every six months, 1% (*n* = 1) thought they would have sufficient information following an initial safer conception visit and 3% (n = 4) were undecided or missing. Again, results did not differ by gender.

**Fig. 2:**
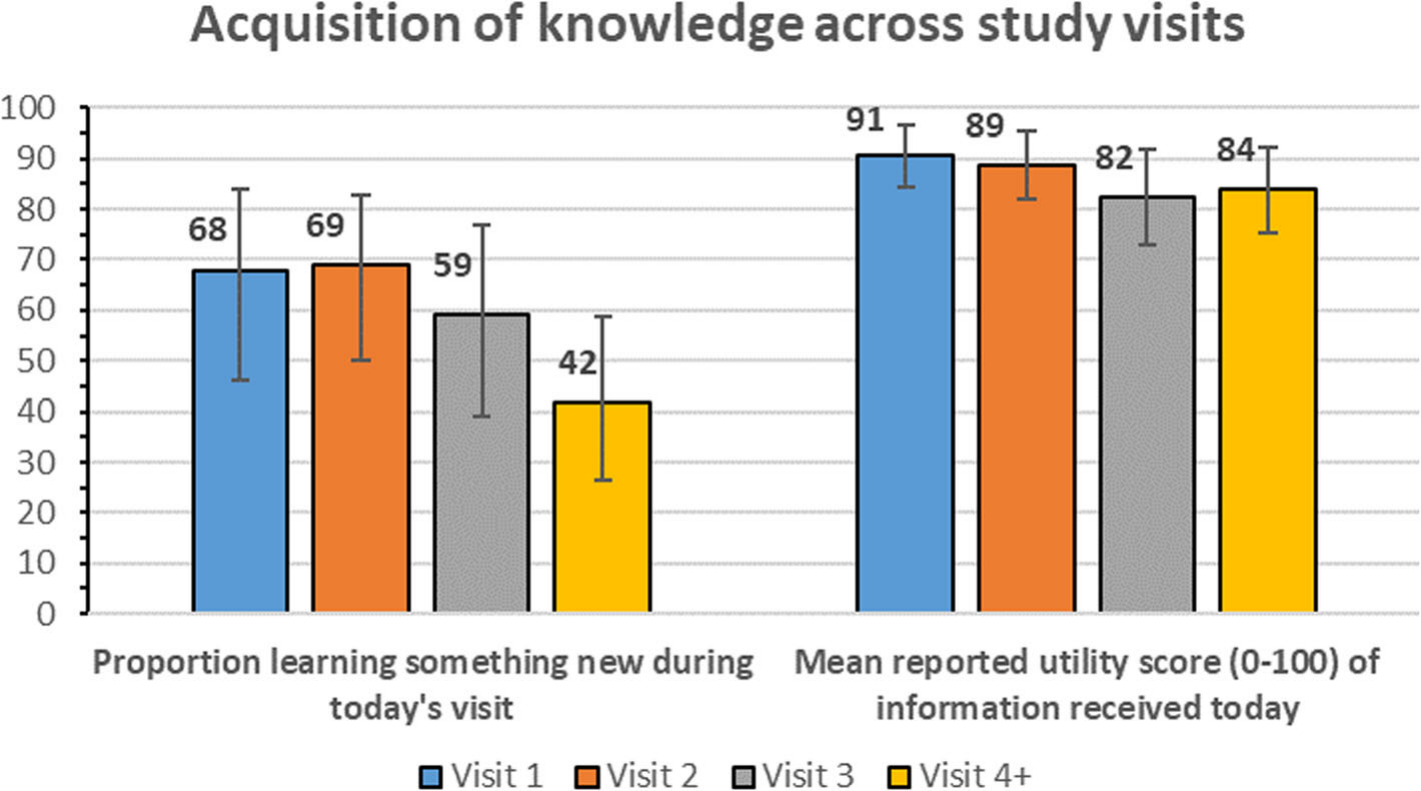
Perceived value and knowledge acquisition across visits among safer conception clients, Johannesburg, South Africa (2016–2017)

In terms of knowledge acquisition, 58% of all clients (*n* = 70) reported learning something new during the current visit, with a trend towards declining levels of new knowledge learned over time (Fig. 2, *p* = 0.09). The mean reported utility score for information received during the visit was largely constant over time (*p* = 0.43). Similarly, no differences were observed across genders. When considering thresholds, 79% reported their current visit to have been highly useful, 18% moderately useful and 3% reported low utility.

On the day of their participation in the process evaluation, 58% of the clients had been seen by a nurse vs. 42% by the doctor. Differences between provider types were observed in terms of clients reporting that they had learned something new during their completed visit, with 48% vs. 73% (*p* = 0.01) reporting that they had learned something new during their visit with the nurse as compared to the doctor. Perceived utility of the visit was high among clients seen by both providers, but higher among clients seen by the doctor (90% perceived the visit to be highly useful, 8% moderately useful and 2% of low utility) as compared to a nurse (71% highly useful, 25% moderately useful and 4% low utility, *p* = 0.04).

### Knowledge and self-efficacy to utilize safer conception methods

**Fig. 3:**
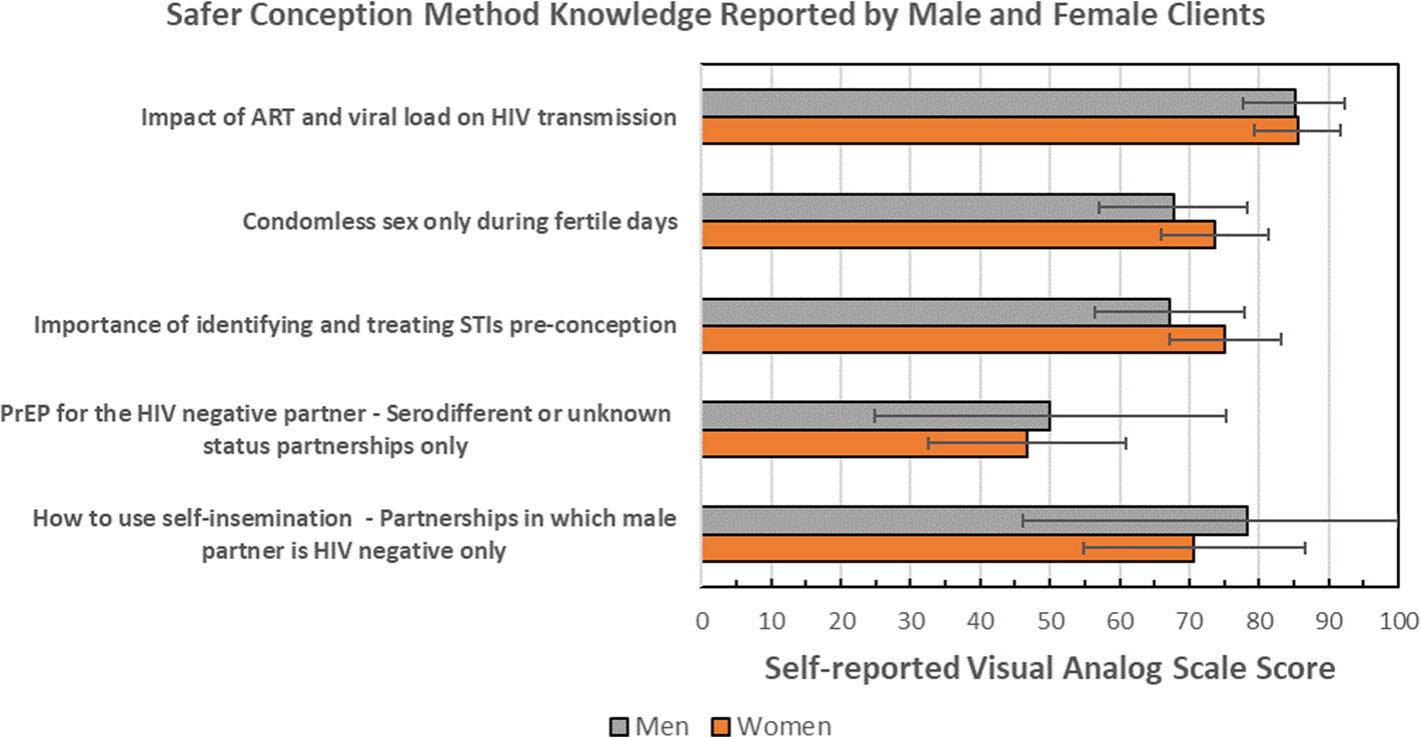
Reported safer conception method knowledge among men and women actively engaged in safer conception care in Johannesburg, South Africa (2016–2017)

Self-rated knowledge of safer conception methods was generally high, though there were variations across safer conception strategies (Fig. 3). Among the strategies relevant to all partnerships, within-individual rankings indicated that awareness of the impact of ART and viral load on HIV transmission was the strategy for which the most clients reported the greatest understanding, followed by condomless sex limited to the most fertile days and treating STIs prior to conception. Overall, 80% reported high levels of perceived knowledge of the impact of ART and viral suppression on HIV transmission, 67% perceived high knowledge of the importance of STI screening and 56% reported high levels of knowledge regarding limiting condomless sex to days of peak fertility. Knowledge of how to use self-insemination was high among 57% of men and women in relationships in which the male partner was HIV-negative. A high level of knowledge about PrEP as a safer conception strategy was reported by 34% of those in HIV sero-different partnerships (24% reported moderate knowledge, 42% low knowledge). Knowledge of safer conception methods was comparable between men and women, and by HIV status (results not shown). In general, reported levels of knowledge were steady across visits completed, with the exception of knowledge around ART and viral suppression which increased from a mean score of 84 (sd 5.3) after the first visit to 95 (sd 1.7) among those completing a fourth visit or greater (*p* = 0.049).

**Fig. 4:**
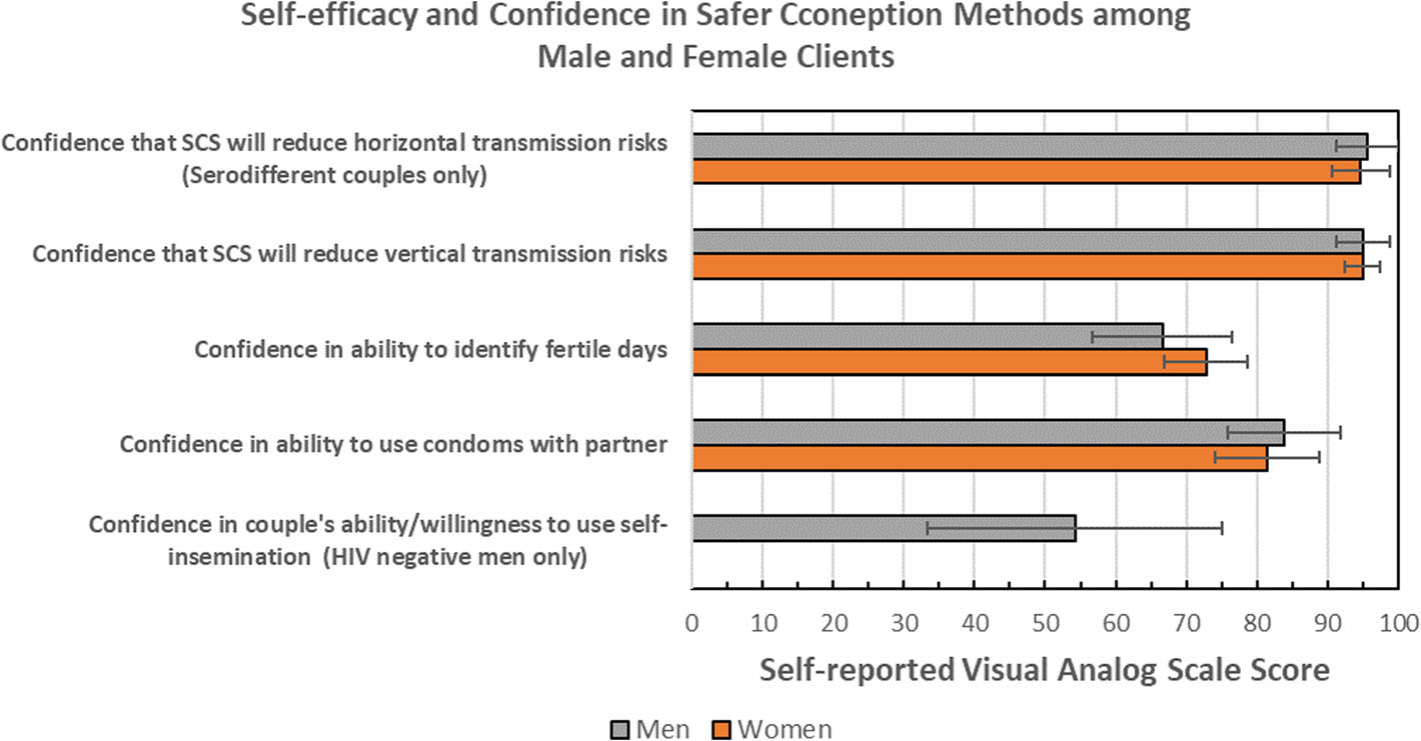
Reported confidence of clients in safer conception methods and their ability to use safer conception methods, Johannesburg, South Africa (2016–2017)

In terms of perceived self-efficacy to identify the most fertile days, the mean score among women and men was 73 (sd 26) and 67 (sd 32) respectively (*p* = 0.26) (Fig. 4); 54% overall reported a high self-confidence score. Confidence in ability to use condoms with a partner was similar between women and men (mean score of 81 [sd 32] vs. 84 [sd 25], *p* = 0.68), and scores were non-statistically significantly higher among those who had ever attended the service as a couple vs. always attending alone at the time of the interview (87 (sd 25) vs. 77 (sd 35), *p* = 0.08). Within-client rankings supported the notion of a higher self-efficacy to use condoms over identification of fertile days, as over 2.4 times more respondents reported higher self-efficacy scores in terms of condom use vs. fertile day identification. Among HIV-negative men utilizing the service, 43% (*n* = 6/14) reported a high score around confidence to correctly use self-insemination techniques to conceive. Differences in confidence to use the various safer conception methods across study visits were explored, but no differences in self-confidence across visits completed were observed (results not shown).

Men and women were both confident that safer conception practices would reduce the risk of vertical transmission to a child should they become pregnant (Fig. 4). Similarly, men and women in sero-different partnerships reported high confidence that engagement in safer conception services would reduce their risks of horizontal HIV transmission.

## Discussion

Implementation lessons learned from this safer conception demonstration project can provide insights into service delivery scale-up in South Africa and beyond. Acceptability and perceived value and utility of services were high among safer conception clients, supporting the expansion of safer conception models if demonstrated to be effective at reducing HIV horizontal and vertical transmission risks. Importantly, acceptability and perceived value were observed equally among men, a population often hard to reach and engage in care. Perceived knowledge and self-efficacy to use safer conception methods were also high among clients utilizing the service, however there were no trends suggestive of increasing knowledge or self-confidence to use safer conception methods across the number of visits completed, which has implications for the recommended frequency of safer conception visits and counseling approach.

Despite client preferences to attend safer conception services monthly or bi-monthly until conception, and the high perceived utility of services across visits, the proportion of clients receiving new information at each visit declined across subsequent visits. Reconciling client preferences for frequent engagement in safer conception care, with apparent declines or plateaus in the return of new information, knowledge and self-efficacy to use methods will be critical in planning for the scale-up of safer conception services. Perceived value and preferences for service delivery among clients must be balanced alongside resource constraints and the need to propose a care model that is both evidence-based and sustainable. Alternative models could test an initial visit with a nurse, with subsequent counselor or peer-led follow-up visits to provide on-going support once critical knowledge transfer has occurred.

As differentiated care models for HIV are being brought to scale in Southern Africa, these models should address safer conception needs. Differentiated care models to-date have focused primarily on adjusting service delivery for virally suppressed patients on ART [25, 26]. Emphasis has been given to reducing the patient burden of frequent clinic visits, while reducing the burden on the healthcare system through task-shifting of care responsibilities to less skilled healthcare workers [27, 28]. In the case of safer conception, however, service delivery needs may conversely require a short-term intensification of services during the periconception period. Moreover, among clients interested in safer conception services, a one-size fits all approach may not be the most efficient or effective strategy. Clients’ health, couples’ sero-status and safer conception method choice should guide service delivery approaches. For example, a sero-same, fully-disclosed couple that are both on ART and virally suppressed may be seen initially to screen for STIs and monitor viral load, but then return to adherence club care or other routine care. On the other hand, for patients with untreated STIs, unsuppressed viral loads or for whom partners have unmet healthcare needs (e.g. HIV testing, treatment, etc.), more sustained contact with safer conception providers may be necessary.

Although safer conception care received from a doctor was perceived to be of a greater utility by clients, services were highly valued by both clients seen by a doctor and a professional nurse. A doctor-driven approach is not scalable in resource limited settings. However, the perception or reality, of reduced service utility and knowledge acquisition among clients seen by professional nurses is important to note, as differentiated safer conception models may utilize lower skilled cadres of nurses or HIV lay counsellors for service delivery. In these cases, decline in perceived utility or benefit of services should be monitored.

Encouragingly, we found perceived knowledge and self-efficacy around utilization of safer conception methods to be high among safer conception clients. Although baseline knowledge and self-efficacy were not measured, knowledge of methods was higher than that reported in other studies, including data collected earlier at this site prior to implementation of safer conception service delivery [13, 29, 30]. These findings suggest that engagement in a safer conception service may have had a positive impact on patient-level understanding of safer conception methods and utilization. Similar to findings from other studies, these data indicate room for improvement in safer conception education around self-insemination and PrEP [10, 11, 31]. Lower PrEP knowledge in this cohort was likely due to the late introduction of PrEP at the service delivery site and a larger emphasis on PrEP counseling only for clients perceived to be most likely to benefit, given that in the case of viral suppression of the HIV-positive partner there is limited additional benefit of adding PrEP [32]. Previous analyses have demonstrated that when patients are given a full range of safer conception options, they may choose methods that are sub-optimal for their individual situation [20]. Finding a balance between ensuring clients are empowered with sufficient knowledge to make informed choices that match their preferences, without overwhelming clients with too wide of an array of options, some of which may be less optimal for their given situation, is an important consideration for safer conception models and provider clinical support tools. In the current reality of “U=U” (“undectable” viral load equals “untransmissable”) [33], the need for extensive safer conception strategies may often be limited, however data demonstrate that an undetectable viral load among clients trying to conceive should not be assumed [13, 19].

This analysis has several limitations. The small and cross-sectional sample makes comparisons over time more difficult. A longitudinal design following changes within individuals over time would give better insights into the trajectories of knowledge transfer and self-efficacy development and utility over time. However, we intentionally sampled clients after completing a range of study visits to explore the impact of duration of safer conception participation on utility, knowledge and self-efficacy. Still, those not retained in the service or lost to follow-up may have perceived less value in the service and would be underrepresented in the sample. Similarly, HIV-negative clients not utilizing PrEP, may not have visited the clinic as frequently as their HIV-positive partners and thus may be similarly underrepresented. Despite these limitations, the sample for the process evaluation was representative of the cohort, increasing confidence that the findings were not merely the product of a biased sample. Adolescents 13–17 years old were not included in the safer conception research but have SRHR needs which are often unaddressed. Inclusion of young men and women in safer conception counseling and youth-friendly community-based demand generation for services will be critical to ensure that safer conception care addresses the HIV prevention needs and long-term reproductive planning of adolescents. Additionally, the service was provided by a small number of dedicated safer conception providers. Comparisons across providers should be viewed with this in mind. General acceptability and perceived value may decrease if providers are less motivated to assist patients in achieving their reproductive goals within an integrated service. Finally, VAS can be difficult to interpret and comparisons across groups should be interpreted with caution [24]. In addition to reporting the absolute scale scores, we have included information for key variables regarding item ranking within individuals, as well as the proportion reporting scores at key performance thresholds.

Information from clients attending safer conception care are scarce and despite limitations, these data provide important insights into the perceived value of services, as well as safer conception knowledge and self-efficacy among attendees. Studies further investigating changes in knowledge and skills development within clients over time may help to refine optimal service delivery schedules for patients. These data suggest, however, that many individuals may receive sufficient benefits from a single or limited number of counselling sessions, though desire for more long-term engagement is common, and whether a more limited approach would result in sustained knowledge and practice is not known. Other approaches such as information leaflets that can be taken home and phone-based applications to track fertility may be able to reduce healthcare provider burden following the initial consultation session.

## Conclusions

Differentiated HIV care models will need to consider how to support the reproductive rights of individuals and accommodate their safer conception needs. Specific models for individuals and couples affected by HIV and trying to become pregnant may take various forms. Some patients will require more intensive services based on their clinical circumstances. However, many couples trying to conceive may potentially receive a ‘lighter touch’ approach, while still minimizing HIV transmission risks. Optimal and evidence-based differentiated care models, particularly in the ‘treat all’ era, are necessary for safer conception scale-up in South Africa and across the region.

A French translation of this article has been included as Additional file 1 (see Additional file 1).

A Portuguese translation of the abstract has been included as Additional file 2 (see Additional file 2).

## Additional files

Additional file 1: Translation of this article into French (PDF 801 kb)

Additional file 2: Translation of the abstract of this article into Portuguese (PDF 120 kb)

## Abbreviations

ANOVA: Analysis of variance statistics
ART: Antiretroviral treatment
HIV: Human immunodeficiency syndrome
PrEP: Pre-exposure prophylaxis
SRHR: Sexual and reproductive health and rights
STI: Sexually transmitted infection
UNAIDS: The Joint United Nations Programme on HIV/AIDS
VAS: Visual analog scales
