# Supplementary material for: Clients’ experiences utilizing a safer conception service for HIV affected individuals: implications for differentiated care service delivery models
Source: Reprod Health. 2019 May 29;16(Suppl 1):65. doi: 10.1186/s12978-019-0718-5 (PMC6538560; doi:10.1186/s12978-019-0718-5)
Supplement: Supplementary file 1 — Translation of this article into French (PDF 801 kb) [file 12978_2019_718_MOESM1_ESM.pdf]

Reproductive Health 2019, 16 (Suppl 1) [translated from the original publication]

Expériences des bénéficiaires d'un service de conception plus sûre destiné aux personnes touchées par le VIH : Conséquences pour les modèles de prestations de services de soins différenciés

Sheree Schwartz[1,2*], Natasha Davies[2], Nicolette Naidoo[2], Diantha Pillay[2], Nokuthula Makhoba[2], Saiqa Mullick[2]

[1] Department of Epidemiology, Johns Hopkins School of Public Health, Baltimore, MD, USA
[2] Wits Reproductive Health Institute, University of the Witwatersrand, Johannesburg, South Africa

*Auteur correspondant : Dr. Sheree Schwartz, 615 N. Wolfe St, E7644, Baltimore, MD, USA, 21205. E-mail : sschwartz@jhu.edu

Adresses électroniques :
Sheree Schwartz: sschwartz@jhu.edu
Natasha Davies: davies@anovahealth.co.za
Nicolette Naidoo: NNaidoo@wrhi.ac.za
Diantha Pillay: DPillay@wrhi.ac.za
Nokuthula Makhoba: NMakhoba@wrhi.ac.za
Saiqa Mullick: SMullick@wrhi.ac.za

RÉSUMÉ

Contexte : Les services de conception plus sûre favorisent la santé reproductive et les droits des familles, tout en minimisant les risques de transmission du VIH entre les partenaires qui tentent de concevoir un enfant, ainsi que les risques de transmission verticale. Cependant, les données concernant la mise en oeuvre de ces services, notamment l'expérience des personnes ayant recours à des services de conception plus sûre en Afrique subsaharienne, sont limitées.

Méthodes : En juin 2015, le centre médical Hillbrow Community Health Centre, à Johannesbourg, en Afrique du Sud, a commencé à proposer des services de conception plus sûre aux personnes et aux couples touchés par le VIH. Entre avril 2016 et août 2017, un sous-échantillon stratifié de bénéficiaires de services de conception plus sûre a été recruté pour participer à une enquête transversale. Cette enquête visait à évaluer la façon dont ces personnes percevaient la valeur et l'acceptabilité des services, ainsi que leurs connaissances

acquises en matière de conception plus sûre et l'auto-efficacité qu'ils en retiraient personnellement. Des échelles visuelles analogiques de 0 à 100 ont été utilisées pour mesurer les expériences des bénéficiaires. Les scores ont été classés de la façon suivante : acceptation/valeur/connaissances/auto-efficacité faibles pour des scores < 50, moyennes pour des scores compris entre 50 et 79 et élevées pour des scores ≥ 80. Des comparaisons inter-scores ont été faites entre les différentes visites de conception plus sûre auxquelles les bénéficiaires se sont rendus.

Résultats : Parmi 692 personnes ayant recours à des services de conception plus sûre, 120 (17 %) ont été échantillonnées pour évaluer les processus. Les caractéristiques des participants de ce sous-échantillon étaient similaires à celles des participants de la cohorte globale. Les bénéficiaires ont donné une note moyenne ≥ 90 points pour chaque question visant à évaluer l'acceptabilité des services et 96 % (114/119) d'entre eux ont indiqué qu'ils trouvaient que le suivi régulier dont ils avaient bénéficié en matière de conception plus sûre jusqu'à la conception était d'une grande valeur (scores ≥ 80). 58 % (n=70) des bénéficiaires ont déclaré avoir appris quelque chose de nouveau pendant la visite à laquelle ils se sont rendus le jour de l'enquête, même si l'acquisition de nouvelles informations tendait à diminuer au fil des visites (p=0,09). En ce qui concerne les stratégies de conception plus sûre, 80 % des bénéficiaires ont signalé avoir acquis un niveau de connaissances élevé sur l'impact du traitement antirétroviral (TAR) et de la suppression de la charge virale sur la transmission du VIH, 67 % ont signalé avoir acquis un niveau de connaissances élevé sur l'importance du dépistage des IST et 56 % sur la limitation des rapports sexuels sans préservatif aux jours présentant un pic de fertilité. Enfin, 34 % ont déclaré avoir acquis un niveau de connaissances élevé sur la prophylaxie pré-exposition (PrEP)

dans les relations sérodifférentes. L'auto-efficacité était variable selon les méthodes de conception plus sûres et elle était similaire entre les différentes visites de l'étude.

Conclusions : Les bénéficiaires ont trouvé que leurs visites dans les structures des programmes de conception plus sûre étaient d'une grande valeur et ils ont déclaré préférer un suivi régulier jusqu'à la conception. Toutefois, nous avons observé une stagnation dans les connaissances et l'auto-efficacité acquises au cours des visites suivant la visite initiale. Des services plus intensifs peuvent convenir à certains bénéficiaires selon le contexte clinique, mais de nombreux couples sont susceptibles de correspondre à une approche plus « légère », qui minimise tout de même les risques de transmission du VIH.

Mots clés : Conception plus sûre, VIH, prévention du VIH, soins différenciés, couples sérodifférents, mise en oeuvre

CONTEXTE

Des efforts conjugués de prévention du VIH, notamment l'accès à un traitement antirétroviral (TAR) pour les personnes vivant avec le VIH, doivent être réalisés à grande échelle pour modifier la trajectoire de l'épidémie de VIH [1]. C'est dans ce but que l'ONUSIDA a mis en place un programme ambitieux, les cibles 90-90-90, ainsi que, plus récemment, une feuille de route sur la prévention du VIH, [2], le but étant d'en finir avec l'épidémie de VIH d'ici 2020 [3]. Cependant, même avec une disponibilité plus importante des traitements en Afrique subsaharienne, certaines populations présentent encore des risques élevés d'infection par le VIH et de transmission du virus [4]. Il existe peu de modèles de prestations de services testés à grande échelle fournissant des services complets répondant aux besoins spécifiques de ces

groupes à risque, notamment les couples infectés par le VIH essayant de concevoir un enfant [5-7].

Les individus et les couples infectés par le VIH qui tentent de concevoir un enfant, notamment ceux impliqués dans des relations sérodifférentes, dans lesquelles l'un des partenaires est séropositif pour le VIH et l'autre est séronégatif pour le VIH, présentent des besoins spécifiques de prévention contre le VIH en raison de leurs objectifs de conception et ils doivent être soutenus dans le cadre de l'accès universel à la santé et aux droits sexuels et reproductifs (SDSR) [8]. Les études montrent systématiquement que les gens vivant avec le VIH désirent généralement avoir des enfants, mais manquent d'informations sur la façon de concevoir de manière plus sûre [9-11]. Par conséquent, les couples infectés par le VIH font souvent passer les tentatives de conception d'un enfant avant l'amélioration de leur état de santé, ce qui est pourtant primordial pour réduire les risques de transmission du VIH et d'infection par ce virus [12-14].

Les services de conception plus sûre présentent une approche globale pour promouvoir les SDSR des familles, tout en minimisant les risques de transmission du VIH entre les partenaires qui tentent de concevoir un enfant, ainsi que les risques de transmission verticale, c'est-à-dire de transmission des mères infectées par le VIH à leurs enfants [15]. Parmi les méthodes combinées de prévention contre le VIH utilisées pour parvenir à une conception plus sûre dans les populations à faibles ressources, citons l'instauration immédiate d'un TAR pour les partenaires séropositifs, la surveillance des charges virales et la confirmation de la suppression de la charge virale pré-conception, le dépistage et le traitement des infections sexuellement transmissibles (IST), l'éducation relative aux dates d'ovulation estimées et aux pics de fertilité

pour déterminer les périodes propices aux relations sexuelles sans préservatif ou à l'auto-insémination à l'aide d'une seringue sans aiguille et l'initiation à la prophylaxie pré-exposition (PrEP) pour les partenaires séronégatifs pour le VIH [16].

Il est de plus en plus admis que les besoins en matière de traitement et de prévention pour le VIH diffèrent selon les individus et les populations. Il est également reconnu que des modèles de prestations de services différenciés peuvent permettre d'atteindre des objectifs de traitement ambitieux en attribuant efficacement les ressources et en fournissant des soins adaptés aux préférences des patients [17]. Les modèles de soins différenciés tiennent compte du lieu et du moment auxquels les services sont fournis, des prestataires de service et de l'ensemble des soins proposés [18]. Les soins de conception plus sûre représentent en eux-mêmes un modèle de soins différenciés dans la mesure où ils fournissent un ensemble de services adaptés aux besoins spécifiques des couples infectés par le VIH qui souhaitent concevoir un enfant.

Bien que les demandes de services de conception plus sûre aient fortement augmenté ces dernières années, peu de ces services de soins primaires ont été introduits en Afrique subsaharienne et les données concernant leur mise en oeuvre commencent seulement à être disponibles [19-21]. Les données concernant l'expérience des bénéficiaires de services de conception plus sûre ne sont pas encore disponibles. Et pourtant, ces données sont primordiales pour évaluer l'acceptabilité et l'impact potentiel des services de conception plus sûre, ainsi que pour optimiser les modèles de prestations de services dans le cadre d'une mise en oeuvre à plus grande échelle. L'objectif de cette étude est d'évaluer l'acceptabilité, la valeur perçue, les connaissances acquises et l'auto-efficacité liées à ces services chez des couples

infectés par le VIH bénéficiant de services de conception plus sûre à Johannesbourg, en Afrique du Sud.

MÉTHODES

Définition de l'étude et population

Des services de conception plus sûre ont été mis en place au sein du département clinique de TAR du centre médical Hillbrow Community Health Centre en juin 2015. Il s'agissait d'un projet de démonstration visant à évaluer la faisabilité, l'acceptabilité et l'efficacité des services de conception plus sûre. Le programme de TAR est suivi par plus de 21 000 patients séropositifs pour le VIH dans le centre urbain densément peuplé de Johannesbourg. Le service de conception plus sûre est un service autonome basé dans le département clinique de TAR, qui accueille des personnes recommandées venant de différents points d'entrée dans le centre de santé communautaire, ainsi que de cliniques à proximité. Les points d'entrée dans le centre de santé communautaire sont, notamment, le département clinique de TAR, la structure de planning familial, le service de dépistage et de conseils sur le VIH, les services de soins primaires généraux, le programme de circoncision médicale masculine et les services itinérants. Toute demande est généralement motivée par des discussions sur la santé, des affiches, des recommandations de conseillers et de cliniciens aux différents points d'entrée et le bouche à oreille.

Les services de conception plus sûre comprennent des conseils et un test de dépistage du VIH, le dépistage des IST et du cancer du col de l'utérus, l'instauration d'un TAR et son suivi, la surveillance de la charge virale, des explications sur les périodes de fertilité et les moments

propices aux rapports sexuels sans préservatif, des instructions sur l'auto-insémination et la PrEP, et un soutien pour l'annonce de sa séropositivité à son partenaire. La PrEP était initialement disponible sur ordonnance dans les pharmacies privées mais elle est désormais disponible dans le département clinique et délivrée au cas par cas en raison de l'augmentation de sa disponibilité dans le secteur de la santé publique du pays. Les services de conception plus sûre sont supervisés par du personnel infirmier, sous la surveillance d'un médecin, qui dispense également des soins. Un conseiller non professionnel de santé propose également des conseils et des tests de dépistage du VIH. Les bénéficiaires de ces services ont été vus tous les 1 à 2 mois. Les consultations initiales duraient environ 45 minutes, avec des visites de suivi généralement comprises entre 15 et 30 minutes.

Les hommes et femmes éligibles pour recevoir ces services et participer à la recherche devaient être âgés d'au moins 18 ans, être impliqués dans une relation dans laquelle au moins l'un des partenaires était séropositif pour le VIH, avoir l'intention de tenter de concevoir un enfant dans les six prochains mois et ne pas avoir reçu de diagnostic d'infertilité non résolu dans le passé.

Conception de l'étude

Le projet de démonstration se base sur l'observation d'une cohorte dans laquelle les bénéficiaires sont informés des stratégies de conception plus sûre et, en accord avec leur prestataire de soins cliniques de conception plus sûre, choisissent les stratégies de conception plus sûre qui leur conviennent. Les services commencent par un test de dépistage du VIH et des IST, puis s'orientent différemment selon les préférences du patient, le statut sérologique du couple et l'implication du partenaire. Le suivi des critères d'évaluation primaires de l'efficacité

des services de conception plus sûre, notamment la survenue d'une grossesse et la transmission du VIH, sera présenté ultérieurement.

Dans le cadre plus large du projet de démonstration des services de conception plus sûre, nous avons cherché à évaluer les résultats liés à leur mise en oeuvre en réalisant une évaluation quantitative des processus, dans le but d'obtenir des informations sur la réussite et les difficultés des mécanismes d'intervention, et d'informer de la possibilité d'envisager une mise en oeuvre plus vaste de ces services. Les principaux éléments évalués étaient l'acceptabilité des prestations de services, la valeur perçue et les connaissances acquises, ainsi que l'auto-efficacité à mettre en oeuvre les stratégies de conception plus sûre.

L'évaluation a été menée dans un sous-échantillon de bénéficiaires de services de conception plus sûre. Les personnes participant à l'étude sur la conception plus sûre et ayant bénéficié de services de conception plus sûre entre avril 2016 et août 2017 ont été ensuite contactés et se sont vus proposer de participer à l'évaluation des processus à la fin de leur visite de conception plus sûre. Dans la mesure où le but de l'évaluation était d'évaluer l'expérience des patients plutôt que l'efficacité ou l'efficience, un échantillon de 120 personnes (devant représenter entre 15 et 20 % de la cohorte) a été ciblé. Un échantillonnage stratifié a été réalisé de façon à ce qu'au moins 25 bénéficiaires soient représentés parmi ceux qui effectuaient une première, une deuxième, une troisième, une quatrième visite ou plus afin qu'un large éventail d'expériences soit représenté parmi les bénéficiaires de services de conception plus sûre. Le comité d'éthique de la recherche avec des êtres humains de l'université de Witwatersrand (protocole *M150146*) a veillé au respect de l'éthique. Tous les participants ont rempli un formulaire de consentement éclairé.

Collecte des données et analyses

Les données d'évaluation des processus ont été collectées lors d'un sondage réalisé en une seule fois et géré par l'enquêteur. Le sondage a été réalisé sur une tablette et capturé dans REDCap, une application Web sécurisée conçue pour la capture et la gestion de données [22]. Il a été mené dans un espace privé, en insistant sur la confidentialité des réponses. Les entretiens ont duré environ 15 minutes, durant lesquels des échelles visuelles analogiques (EVA) ont été souvent utilisées pour permettre aux bénéficiaires des services de noter leur expérience sur une échelle de 0 à 100 [23].

Par ailleurs, des analyses descriptives ont évalué l'acceptabilité, la valeur perçue, les connaissances acquises et l'auto-efficacité des services de conception plus sûre. L'acceptabilité des services de conception plus sûre a été mesurée à l'aide d'une EVA évaluant si le patient était à l'aise lorsqu'il était vu avec son partenaire, s'il acceptait facilement le temps passé lors des visites et comment il percevait la politique de respect de la vie privée/confidentialité du service. L'acceptabilité et la valeur perçue ont été évaluées de façon plus approfondie à l'aide d'une EVA notant la valeur perçue pour les patients ayant régulièrement recours aux services de conception plus sûre jusqu'à la fin de la grossesse, leurs préférences en matière de fréquence des visites de conception plus sûre, la proportion des patients ayant appris quelque chose de nouveau pendant leurs visites et une EVA évaluant la notion d'utilité des informations reçues pendant les visites. Les différents éléments liés à l'annonce de la séropositivité d'un patient à son partenaire ont constitué des moyens supplémentaires de mesurer l'acceptabilité et la valeur des services évalués par les EVA. La perception des connaissances acquises et l'auto-efficacité résultant de la mise en place des méthodes de conception plus sûres ont été

indiquées par les bénéficiaires à l'aide de la notation par EVA. La confiance des bénéficiaires dans les services pour leur permettre de réduire leurs risques de transmission horizontale et verticale du VIH a également été indiquée. En plus des retours d'informations et des écarts types des scores d'EVA, les participants ont été répartis de la façon suivante selon leur perception : acceptation/valeur/connaissances/auto-efficacité faibles si leurs scores d'EVA étaient < 50, moyennes s'ils étaient compris entre 50 et 79 et élevées s'ils étaient ≥ 80. Ils ont ensuite été classés en fonction de ces niveaux seuil. Des comparaisons ont été faites par sexe et entre les différentes catégories de visites de l'étude (par ex., première, deuxième, troisième, quatrième visite ou plus) à l'aide de statistiques basées sur le chi carré afin de comparer les variables catégorielles et les tests t ou via l'analyse de variance (ANOVA) afin de comparer les scores EVA moyens entre deux, trois ou plusieurs groupes respectivement. Enfin, les scores EVA sur les connaissances acquises et l'auto-efficacité liées aux méthodes de conception plus sûres ont été classés afin de réaliser une analyse de sensibilité visant à comparer les connaissances acquises et l'auto-efficacité entre les différentes méthodes [24]. Par exemple, nous avons évalué le pourcentage de participants ayant signalé que la méthode pour laquelle ils avaient acquis le niveau le plus élevé de connaissances sur la conception plus sûre était la suppression de la charge virale, ou bien l'auto-insémination, ou encore la PrEP, etc. Les analyses ont été effectuées à Stata 14.1 (College Station, Texas).

RÉSULTATS

Parmi les 692 bénéficiaires de services de conception plus sûre, 120 (17 %) ont été échantillonnés pour l'évaluation des processus, dont 78 femmes et 42 hommes. Pour évaluer la représentativité du sous-échantillon, nous avons comparé les caractéristiques des participants

de la cohorte globale à celles des participants à l'évaluation des processus (tableau 1). Les patients participant à l'évaluation des processus étaient comparables à ceux de la cohorte globale.

Les bénéficiaires de services de conception plus sûre participant à l'évaluation des processus s'étaient rendus à trois (médiane) visites de conception plus sûre [écart interquartile (EI) 2-4] au moment du sondage. 21 % (n=25) des participants ont réalisé l'entretien après leur première visite de conception plus sûre, 27 % (n=32) après leur deuxième visite, 22 % (n=27) après leur troisième visite et 30 % (n=36) après leur quatrième visite ou plus.

Acceptabilité et valeur perçue des services de conception plus sûre.

L'acceptabilité du service était élevée. Les participants ont donné un score moyen de 90 points ou plus pour chaque question évaluant l'accessibilité, notamment s'ils étaient à l'aise en étant vus avec leur prestataire de soins en présence de leur partenaire, le temps passé dans le département clinique et le niveau de confidentialité et de respect de la vie privée (figure 1). Les résultats étaient les mêmes entre hommes et femmes. Globalement, 57 % (n=68/120) des personnes participant à l'évaluation des processus s'étaient rendus à au moins une visite de conception plus sûre avec leur partenaire au moment de l'entretien.

Parmi ceux qui n'avaient pas suivi le programme avec leur partenaire, 62 % (n=32/52) ont indiqué que leur partenaire avait l'intention de les accompagner à une prochaine visite. Huit participants n'avaient pas annoncé leur séropositivité pour le VIH à leur partenaire au moment de l'évaluation des processus.

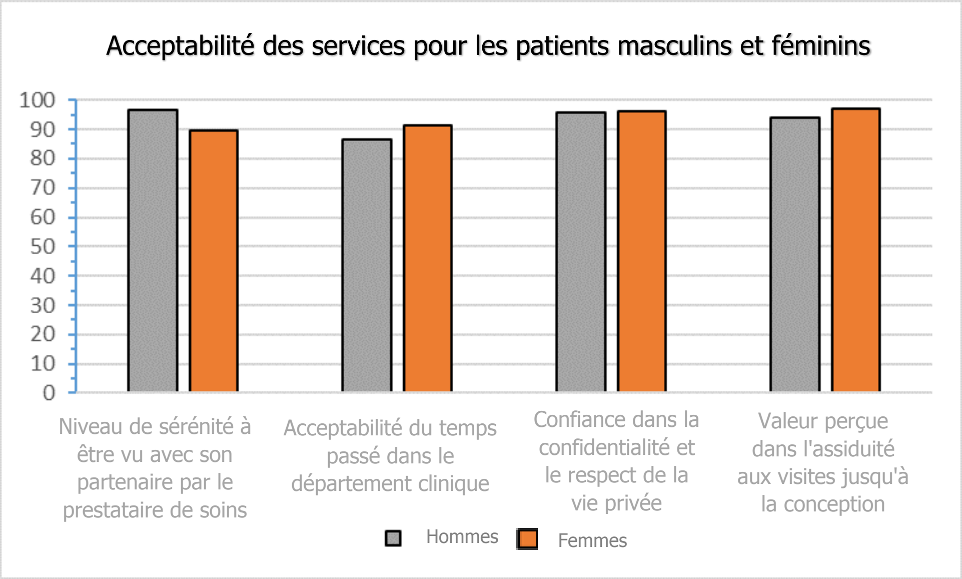

Figure 1 : Acceptabilité des services chez les hommes et les femmes suivant un programme de conception plus sûre à Johannesbourg, en Afrique du Sud (2016-2017)

En termes d'acceptabilité et de valeur spécifiques à l'annonce de leur séropositivité (n=8), 63 % ont trouvé que les conseils qu'ils avaient reçus à propos de cette annonce à des fins de conception plus sûre étaient d'une valeur élevée (25 % d'une valeur moyenne et 12 % d'une valeur faible). 50 % ont indiqué qu'ils étaient peu à l'aise avec les prestataires de soins pendant les discussions sur l'annonce (25 % moyennement à l'aise et 25% très à l'aise), tandis que beaucoup d'entre eux ont déclaré avoir ressenti une faible pression des prestataires de soins pour annoncer leur situation à leur partenaire (75 % une faible pression et 25 % une pression élevée).

Globalement, 96 % (n=114/119) ont indiqué avoir ressenti fortement l'importance de se rendre régulièrement au département clinique de conception plus sûre jusqu'à la conception (score EVA ≥ 80). Cette importance ressentie de l'assiduité était similaire selon le sexe (figure 1), ainsi que selon le nombre de visites de conception plus sûre auxquelles les participants s'étaient déjà rendus (résultats non présentés). De même, lorsqu'ils ont été interrogés sur leurs préférences

pour la fréquence des visites, 41 % (n=49) des participants ont déclaré préférer les visites mensuelles, 21 % (n=25) une visite tous les deux mois, 32 % (n=38) une visite tous les trois mois et 2 % (n=3) une visite tous les six mois. 1 % (n=1) pensaient qu'ils auraient suffisamment d'informations en suivant une visite de conception plus sûre initiale et 3 % (n=4) étaient indécis ou absents. Là encore, les résultats étaient les mêmes indépendamment du sexe.

En termes d'acquisition des connaissances, 58 % de tous les participants (n=70) ont indiqué avoir appris quelque chose de nouveau durant la visite actuelle, avec une tendance à acquérir moins de connaissances avec le temps (figure 2, p=0,09). Le score d'utilité moyen indiqué pour les informations reçues durant la visite était très stable dans le temps (p=0,43). De même, aucune différence n'a été observée selon le sexe. En termes de seuils, 79 % des participants ont indiqué que leur visite actuelle avait été très utile, 18 % qu'elle avait été moyennement utile et 3 % qu'elle avait été peu utile.

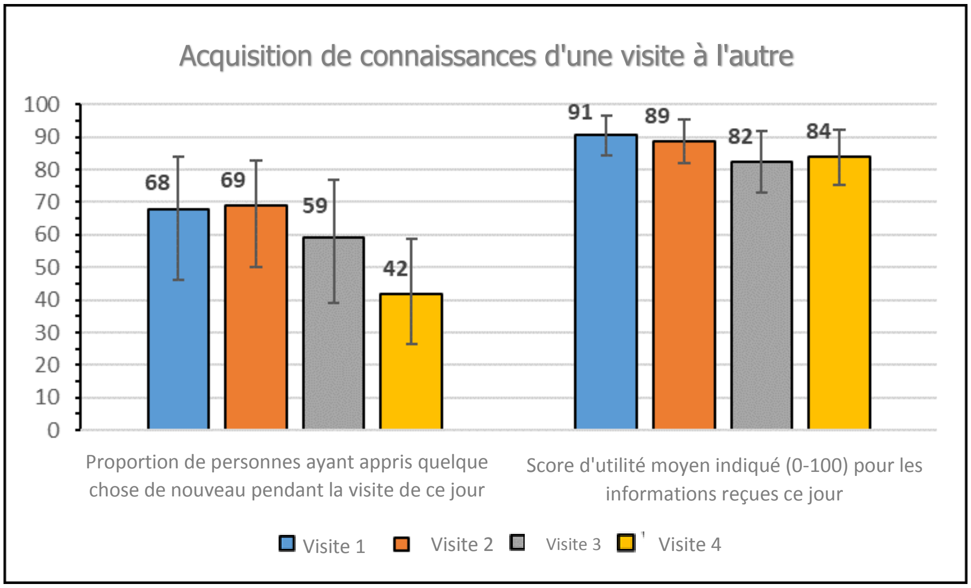

Figure 2 : *Valeur perçue et connaissances acquises par les bénéficiaires de services de conception plus sûre au cours de leurs visites, Johannesbourg, Afrique du Sud (2016-2017)*

Le jour de leur participation à l'évaluation des processus, 58 % des participants avaient été vus par un membre du personnel infirmier et 42 % par le médecin. Des différences ont été observées selon le type de prestataire de soins dans les déclarations faites par les participants expliquant qu'ils avaient appris quelques chose de nouveau durant leur visite, 48 % des participants contre 73 % (p=0,01) déclarant qu'ils avaient appris quelque chose de nouveau pendant leur visite avec le membre du personnel infirmier par rapport au médecin. L'utilité de la visite était perçue comme élevée parmi les participants vus par les deux prestataires de soins mais elle était perçue comme plus élevée chez les participants vus par le médecin (90 % ayant trouvé que la visite était très utile, 8 % qu'elle était moyennement utile et 2 % qu'elle était peu utile) que chez ceux vus par le membre du personnel infirmier (71 % ayant trouvé que la visite était très utile, 25 % qu'elle était moyennement utile et 4 % qu'elle était peu utile, p=0,04).

Connaissances et auto-efficacité liées à l'utilisation de méthodes de contraception plus sûre

Le résultat de l'auto-évaluation des connaissances acquises par les méthodes de conception plus sûres était généralement élevé, malgré des variations selon les stratégies de conception plus sûre (figure 3). Parmi les stratégies pertinentes pour tous les types de couples, le classement entre les individus indiquait que la prise de conscience de l'impact du TAR et de la charge virale sur la transmission du VIH était la stratégie que les participants déclaraient avoir le mieux compris, suivie par les périodes de relations sexuelles sans préservatif limitées aux jours les plus fertiles puis par le traitement des IST avant la conception. Globalement, 80 % des bénéficiaires ont signalé des niveaux élevés de connaissances sur l'impact du TAR et de la suppression de la charge virale sur la transmission du VIH, 67 % ont signalé des niveaux élevés de connaissances sur l'importance du dépistage des IST et 56 % sur des rapports sexuels sans

préservatif limités aux jours présentant un pic de fertilité. Les connaissances relatives à l'auto-insémination étaient élevées chez 57 % des hommes et des femmes vivant en couple et dont le partenaire masculin était séronégatif pour le VIH. 34 % des participants en couple avec un partenaire sérodifférent pour le VIH ont indiqué avoir un niveau élevé de connaissances sur la PrEP comme stratégie de conception plus sûre (24 % ont indiqué un niveau de connaissances moyen et 42 % un niveau faible). Les connaissances sur les méthodes de conception plus sûres étaient comparables entre les hommes et les femmes et selon leur statut sérologique pour le VIH (résultats non présentés). En général, les niveaux de connaissances indiqués étaient stables d'une visite à l'autre, à l'exception des connaissances relatives au TAR et à la suppression de la charge virale, qui augmentaient, passant d'un score moyen de 84 (écart-type 5,3) après la première visite à 95 (écart-type 1,7) chez ceux qui s'étaient rendus à une quatrième visite ou plus (p=0,049).

En ce qui concerne la perception de l'auto-efficacité à identifier les jours les plus fertiles, le score moyen parmi les femmes et les hommes était de 73 (écart type 26) et 67 (écart type 32) respectivement (p=0,26) (figure 4) ; 54 % ont globalement indiqué un score élevé de confiance en soi. La confiance dans la possibilité d'utiliser des préservatifs avec un partenaire était similaire entre les femmes et les hommes (score moyen de 81 [écart type 32] contre 84 [écart type 25], p=0,68) et les scores n'étaient pas particulièrement plus élevés sur le plan statistique chez ceux qui avaient déjà eu recours au service en tant que couple que chez ceux qui y avaient toujours eu recours seuls au moment de l'entretien (87 [écart type 25] contre 77 [écart type 35), p=0,08). Les classements entre les participants ont étayé la notion d'une auto-efficacité plus élevée dans l'utilisation des préservatifs que dans l'identification des jours de fertilité.

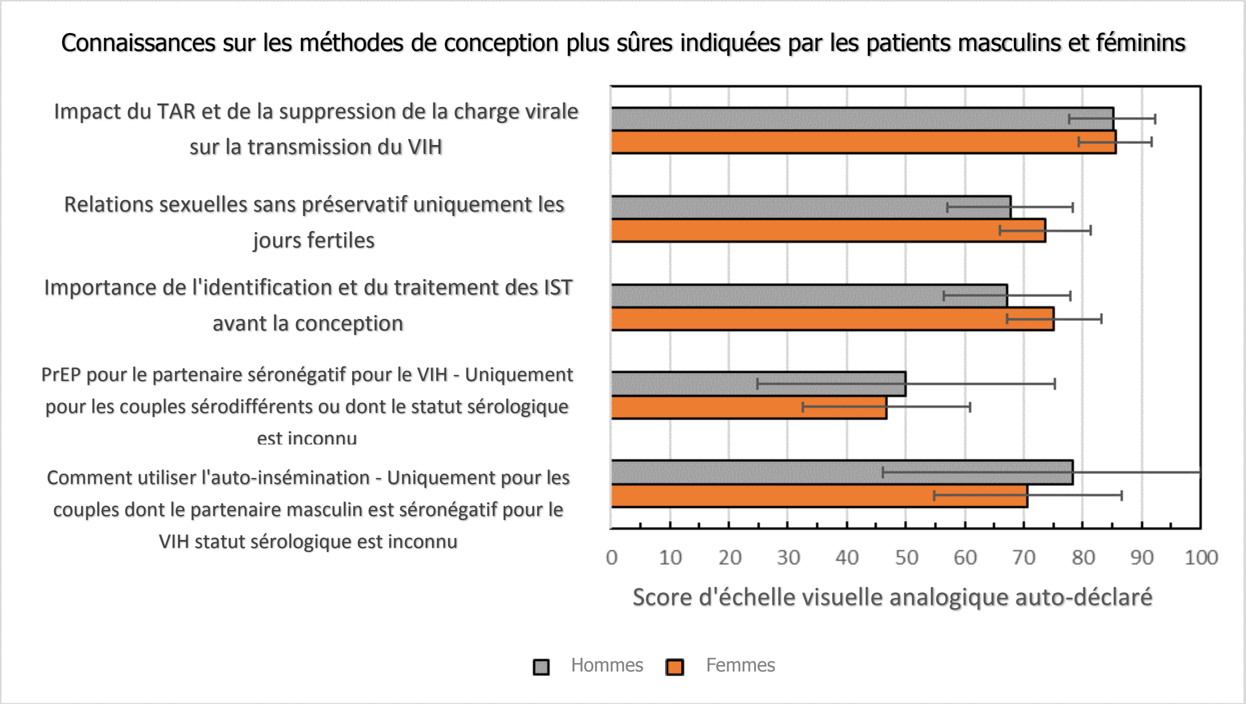

Figure 3 : *Connaissances relatives aux méthodes de conception plus sûres chez des hommes et des femmes activement engagés dans un programme de conception plus sûre à Johannesbourg, en Afrique du Sud (2016-2017)*

En effet, plus de 2,4 fois plus de personnes interrogées ont indiqué des scores d'auto-efficacité plus élevés en termes d'utilisation de préservatifs que d'identification des jours fertiles. Parmi les hommes séronégatifs pour le VIH ayant recours aux services, 43 % (n=6/14) ont indiqué un score élevé en termes de confiance par rapport à l'utilisation correcte des techniques d'auto-insémination pour concevoir un enfant. Les différences de confiance en termes d'utilisation des différentes méthodes de conception plus sûres d'une visite à l'autre ont été étudiées, mais aucune différence en termes de confiance en soi d'une visite à l'autre n'a été observée (résultats non présentés).

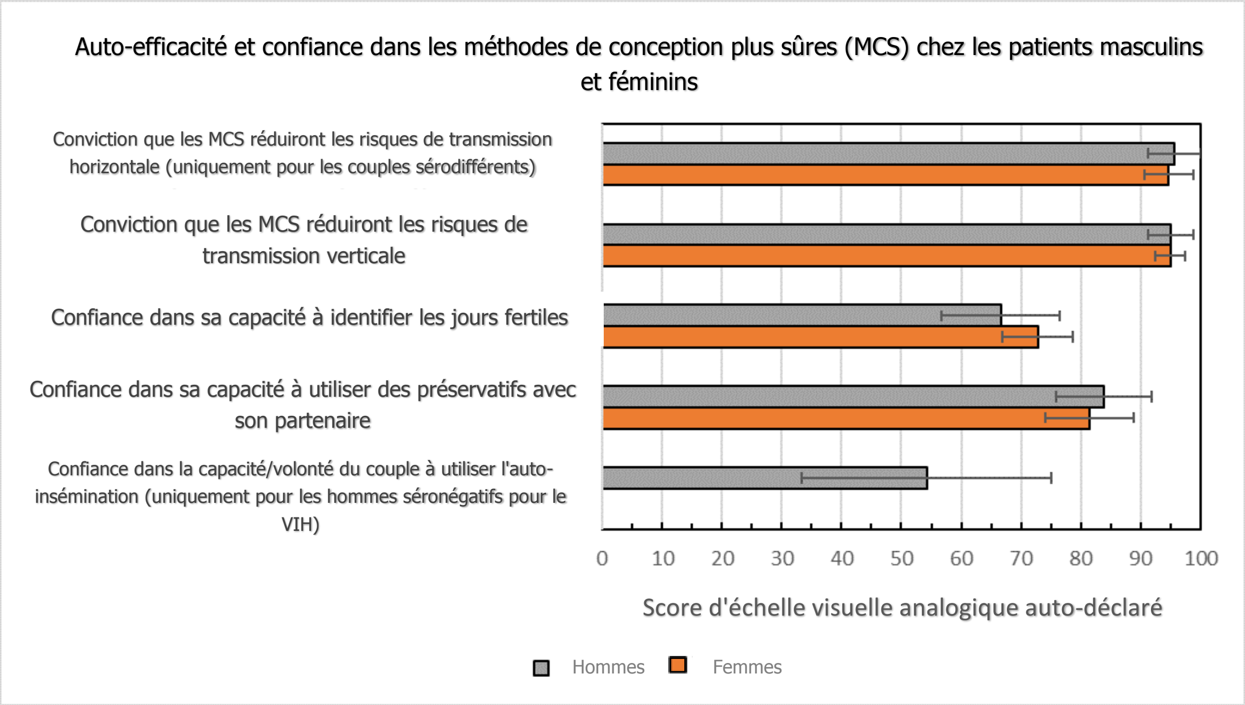

Figure 4 : *Confiance indiquée par les participants en termes de méthodes de conception plus sûres et capacité à utiliser les méthodes de conception plus sûres, Johannesbourg, Afrique du Sud (2016-2017)*

Les hommes comme les femmes pensaient que les méthodes de conception plus sûres réduiraient le risque de transmission verticale à un enfant s'ils devaient en concevoir un (figure 4). De même, les hommes et les femmes en couple avec un partenaire sérodifférent ont indiqué un niveau élevé de confiance dans le fait que leur participation à un programme de conception plus sûre réduirait les risques de transmission horizontale du VIH.

DISCUSSION

Les enseignements relatifs à la mise en oeuvre tirés de ce projet de démonstration des services de conception plus sûre apportent des informations utiles pour la mise en place de services à plus grande échelle en Afrique du Sud et ailleurs. L'acceptabilité, la valeur perçue et l'utilité des

services étaient plus élevées chez les individus participant à un programme de conception plus sûre, confirmant l'intérêt de développer des modèles de conception plus sûre supplémentaires si leur efficacité à réduire les risques de transmission horizontale et verticale du VIH était démontrée. Fait non négligeable, l'acceptabilité et la valeur perçue ont été observées de manière égale chez les hommes, une population souvent difficile à atteindre et à convaincre de participer à des programmes de soins. La perception des connaissances acquises et de l'auto-efficacité à utiliser des méthodes de conception plus sûres était également élevée chez les participants ayant recours aux services de conception plus sûre, même si ces connaissances et cette efficacité n'augmentaient pas avec le nombre de visites effectuées. Ces données ont bien sûr une influence sur les conseils et les recommandations en matière de fréquence des visites de conception plus sûre.

Bien que les participants aient exprimé une préférence pour des visites mensuelles ou tous les deux mois jusqu'à la conception et qu'ils trouvent les services très utiles d'une visite à l'autre, la proportion de participants recevant de nouvelles informations à chaque visite diminuait au fur et à mesure des visites. Le rapprochement des préférences des participants concernant la fréquence des visites de conception plus sûre avec les baisses ou les stagnations observées dans le niveau de nouvelles informations, de connaissances acquises ou d'auto-efficacité à utiliser les méthodes de conception plus sûres sera crucial pour planifier la mise en oeuvre à grande échelle de services de conception plus sûre. La valeur perçue et les préférences indiquées par les participants pour les prestations de services doivent être examinées en prenant en compte les contraintes de ressources et la nécessité de proposer un modèle de soins éprouvé et durable. On pourrait envisager des modèles alternatifs proposant une visite

initiale avec un membre du personnel infirmier, puis des visites de suivi avec un conseiller ou un pair afin de fournir un accompagnement continu une fois le transfert des connaissances primordiales effectué.

Dans la mesure où des modèles de soins différenciés pour le VIH sont mis en oeuvre à grande échelle en Afrique du Sud, ces modèles doivent répondre à des besoins de conception plus sûre. Jusqu'à présent, les modèles de soins différenciés étaient principalement axés sur l'ajustement des prestations de services aux patients dont la charge virale avait été supprimée et qui suivaient un TAR [25, 26]. L'accent était mis sur la réduction, pour les patients, des contraintes liées aux visites fréquentes dans le département clinique, ainsi que sur la réduction, pour le système de soins, des contraintes liées au transfert de responsabilités des soins à des prestataires de soins de santé moins qualifiés [27, 28]. Dans le cas de la conception plus sûre, à l'inverse, les besoins en prestations de services peuvent nécessiter une intensification des services à court terme pendant la période de conception proprement dite. Par ailleurs, pour les participants intéressés par les services de conception plus sûre, une approche universelle ne constituerait probablement pas la solution la plus efficace. Les prestations de services doivent être choisies en fonction de la santé des participants, du statut sérologique des couples et de leur choix d'avoir recours à une méthode de conception plus sûre. Par exemple, un couple présentant le même statut sérologique, dont chaque partenaire connaît le statut de l'autre, suivant tous les deux un TAR et dont la charge virale a été supprimée, peut être vu initialement pour un dépistage des IST et une surveillance de la charge virale, puis revenir à des soins liés à un programme d'observance ou à d'autres soins courants. En revanche, les patients présentant des IST non traitées, des charges virales non supprimées ou pour lesquels le partenaire ne

reçoit pas les soins nécessaires à son état (par ex., test de dépistage du VIH, traitement, etc.), nécessiteraient un contact plus approfondi avec des prestataires de soins de conception plus sûre.

En outre, bien que les participants aient trouvé les soins de conception plus sûre prodigués par un médecin plus utiles, les patient ayant été reçus par un médecin tout comme ceux ayant été reçus par un membre qualifié du personne infirmier ont tous affirmé avoir reçu des soins d'une grande valeur. Une initiative entreprise par un médecin ne peut pas être étendue à des environnements à ressources limitées. Cependant, il est important de noter que les services ont semblé moins utiles et l'acquisition des connaissances moins importante chez les participants ayant été vus par des membres qualifiés du personnel infirmier, que cela corresponde à une réalité ou à un ressenti, car les prestations de services de certains modèles de conception plus sûre différenciés pourraient être réalisées par du personnel infirmier moins qualifié ou des conseillers non professionnels de santé formés pour la prise en charge du VIH. Dans ces cas précis, il faudrait surveiller la perception éventuelle d'une baisse de l'utilité ou des bénéfices tirés des services.

Nous avons cependant trouvé que les participants aux services de conception plus sûre pensaient avoir acquis un niveau élevé de connaissances et d'auto-efficacité dans le cadre de l'utilisation des méthodes de conception plus sûres, ce qui est encourageant. Bien que les connaissances de base et l'auto-efficacité n'aient pas été mesurées, le niveau de connaissance des méthodes était supérieur à celui indiqué dans les autres études, et comprenait les données collectées précédemment sur ce site, avant la mise en oeuvre des prestations de services de conception plus sûre [13, 29, 30]. Ces résultats semblent indiquer que la participation à un

programme de conception plus sûre aurait eu un impact positif sur la compréhension et l'utilisation des méthodes de conception plus sûres par les patients. Ces données, semblables aux résultats d'autres études, indiquent qu'il doit être possible d'améliorer la formation des personnes à la conception plus sûre dans les domaines de l'auto-insémination et de la PrEP [10, 11, 31]. Le plus faible niveau de connaissances dans le domaine de la PrEP dans cette cohorte s'explique probablement par l'introduction tardive de la PrEP sur le site de prestation de services, ainsi que par le fait que la PrEP était conseillée en priorité aux personnes perçues comme étant les plus susceptibles d'en tirer profit. En effet, dans le cas d'un partenaire séropositif pour le VIH dont la charge virale a été supprimée, le bénéfice apporté par l'ajout de la PrEP est limité [32]. Des analyses précédentes ont démontré que lorsque l'on présente un grand choix de méthodes de conception plus sûres aux patients, ils choisissent parfois des méthodes sous-optimales par rapport à leur situation personnelle [20]. Pour établir des modèles de conception plus sûre et mettre en oeuvre des outils d'assistance clinique pour les prestataires de services, il faut donc s'assurer que les patients sont suffisamment informés pour prendre des décisions éclairées en accord avec leur préférences mais ne pas leur proposer un trop grand choix d'options, car certaines seraient sous-optimales par rapport à leur situation. La réalité actuelle du concept « U=U » (charge virale indétectable = non transmission, « undetectable » = « untransmissable » en anglais) [33] réduit souvent la nécessité de mettre en place des stratégies de conception plus sûre étendues. Cependant, les données montrent qu'on ne peut pas compter sur une charge virale indétectable chez les patients tentant de concevoir un enfant [13, 19].

Cette analyse présente toutefois certaines limites. La petite taille de l'échantillon et sa nature transversale rendent les comparaisons plus difficiles dans le temps. Une conception longitudinale suite à des modifications dans le temps chez les individus donnerait une meilleure idée des trajectoires du transfert de connaissances et du développement de l'auto-efficacité ainsi que de l'utilité des services dans le temps. Cependant, nous avons sélectionné volontairement des patients ayant réalisé un certain nombre de visites de l'étude afin d'explorer l'impact de la durée de la participation à un programme de conception plus sûre sur l'utilité des services, les connaissances et l'auto-efficacité. Cependant, ceux qui n'ont pas été maintenus dans le programme ou dont on a perdu la trace pendant le suivi avaient une moins bonne opinion des services et auraient été sous-représentés dans l'échantillon. De même, les participants séronégatifs pour le VIH n'ayant pas recours à la PrEP étaient susceptibles de s'être rendus moins souvent dans le département clinique que leurs partenaires séropositifs au VIH et auraient donc également été sous-représentés. Malgré ces limites, l'échantillon pour l'évaluation des processus était représentatif de la cohorte, ce qui confortait l'idée que les résultats n'étaient pas simplement le produit d'un échantillon biaisé. Les adolescents âgés de 13 à 17 ans n'ont pas été inclus dans l'étude sur la conception plus sûre, mais ils ont des besoins en termes de SDSR qui ne sont souvent pas pris en charge. L'inclusion de jeunes hommes et de jeunes femmes dans les programmes de conseils sur la conception plus sûre et l'apparition d'une demande de services basée sur la communauté et tournée vers les jeunes seront essentielles pour s'assurer que les soins de conception plus sûre répondent aux besoins de prévention du VIH et au planning familial à long terme des adolescents. Par ailleurs, les services ont été fournis par un petit nombre de prestataires de soins de conception plus sûre

spécialisés. Il faut donc comparer les prestataires en gardant cela à l'esprit. L'acceptabilité générale et la valeur perçue peuvent diminuer si les prestataires sont moins motivés à aider leurs patients à atteindre leurs objectifs en matière de reproduction au sein d'un service intégré. Enfin, l'EVA peut être difficile à interpréter et les comparaisons entre les groupes doivent être analysées avec précaution [24]. En plus d'enregistrer la valeur absolue des scores d'échelles, nous avons inclus des informations relatives aux variables clés concernant la classification d'éléments chez les individus, ainsi que la proportion de personnes signalant des scores à des seuils de performances clés.

Les informations fournies par les personnes participant à un programme de conception plus sûre sont rares et malgré ces limites, ces données fournissent des informations importantes sur la façon dont les services sont perçus, ainsi que sur les connaissances des participants en termes de conception plus sûre et sur leur auto-efficacité. Les études qui s'intéressent de plus près aux changements dans le temps en termes de connaissances et de développement des compétences chez les participants peuvent aider à affiner et optimiser les plannings de prestations de services pour les patients. Ces données suggèrent, cependant, que de nombreux individus peuvent tirer suffisamment de bénéfices d'une seule séance ou d'un nombre limité de séances de conseils, bien que le désir de s'engager à plus long terme soit courant. Mais elles ne révèlent pas si une approche plus limitée permettrait des connaissances et une pratique soutenues. D'autres approches telles que les brochures d'informations à emportez chez soi et les applications pour téléphones pour suivre sa fertilité peuvent réduire les contraintes pesant sur les prestataires de soins après la consultation initiale.

Conclusions

Les modèles de soins différenciés pour le VIH devront prendre en compte la façon de soutenir les droits reproductifs des individus et de répondre à leurs besoins en matière de conception plus sûre. Il existe différents modèles spécifiques pour les individus et les couples touchés par le VIH souhaitant concevoir un enfant. Certains patients nécessitent des services plus intensifs, selon le contexte clinique. Toutefois, de nombreux couples essayant de concevoir un enfant sont susceptibles de correspondre à une approche plus « légère », qui minimise tout de même les risques de transmission du VIH. Des modèles de soins différenciés optimaux et éprouvés, alors que la tendance est aux soins universels, sont nécessaires pour étendre la conception plus sûre en Afrique du Sud et dans toute la région.

Abréviations

ANOVA	Analyse de variance (statistiques)
TAR	Traitement antirétroviral
VIH	Virus de l'immunodéficience humaine
PrEP	Prophylaxie préexposition
SDSR	Santé et droits sexuels et reproductifs
IST	Infection sexuellement transmissible
ONUSIDA	Programme commun des Nations Unies sur le VIH/SIDA
EVA	Échelles visuelles analogiques

Concernant ce supplément

Cet article a été publié comme partie de l'ouvrage *Reproductive Health*, Volume 16 Supplement 1, 2019: Effective Integration of Sexual Reproductive Health and HIV Prevention, Treatment, and Care Services across sub-Saharan Africa: Where is the evidence for program implementation?
Le supplément a été publié dans le cadre d'une collaboration entre *Reproductive Health* et *BMC Public Health.* L'intégralité du contenu, avec les versions en français, en portugais et en anglais, est disponible en ligne :
https://bmcpublichealth.biomedcentral.com/articles/supplements/volume-19-supplement-1
et
https://reproductive-health-journal.biomedcentral.com/articles/supplements/volume-16-supplement-1

DÉCLARATIONS

Approbation éthique et accord de participation

Le comité d'éthique de la recherche avec des êtres humains de l'université de Witwatersrand (protocole *M150146*) a veillé au respect de l'éthique. Tous les participants ont rempli un formulaire de consentement éclairé pour participer à la sous-étude.

Accord de publication

Non applicable

Disponibilité des données et matériels

Les données analysées durant l'étude actuelle sont disponibles auprès des auteurs sur demande raisonnable.

Conflits d'intérêts

Les auteurs déclarent ne pas avoir de conflits d'intérêts.

Financement

Le supplément de la revue est rendu possible grâce au soutien généreux du peuple américain via la United States Agency for International Development (USAID) en partenariat avec le Fonds des Nations unies pour la population (FNUAP) et le Programme commun des Nations Unies sur le VIH/SIDA (ONUSIDA).

Les opinions exprimées dans la présente publication sont celles des auteurs et ne reflètent pas nécessairement les politiques officielles de l'USAID, du FNUAP ou de l'ONUSIDA, la mention des

dénominations de ministères ou d'organismes n'implique pas non plus l'aval du gouvernement américain, du FNUAP ou de l'ONUSIDA.

Contributions des auteurs

L'étude a été élaborée par ND, NN, SM et SS. L'étude a été mise en oeuvre par ND, DP et NM. SS a mené l'analyse et tous les auteurs (SS, ND, NN, SM, DP, NM) ont participé à sa rédaction. Tous les auteurs ont lu et approuvé le manuscrit final.

Remerciements

Nous remercions les participants à l'étude d'avoir partagé leurs expériences ainsi que l'équipe de l'étude pour le temps et le dévouement qu'elle y a consacré. Nous remercions également le centre médical Hillbrow Community Health Centre, la ville de Johannesbourg et le ministère sud-africain de la santé pour leur soutien dans ce projet.

RÉFÉRENCES

1. Chang LW, Serwadda D, Quinn TC, Wawer MJ, Gray RH, Reynolds SJ: **Combination implementation for HIV prevention: moving from clinical trial evidence to population-level effects**. *The Lancet infectious diseases* 2013, **13**(1):65-76.
2. UNAIDS: **HIV Prevention 2020 Road Map — Accelerating HIV prevention to reduce new infections by 75%**. Geneva, Switzerland: UNAIDS; 2017.
3. UNAIDS: **90-90-90 - An ambitious treatment target to help end the AIDS epidemic**. Geneva, Switzerland; 2014.
4. UNAIDS: **Prevention Gap Report**. Geneva, Switzerland; 2016.

5. Mmeje O, Titler MG, Dalton VK: **A call to action for evidence-based safer conception interventions for HIV-affected couples desiring children in Sub-Saharan Africa**. *International journal of gynaecology and obstetrics: the official organ of the International Federation of Gynaecology and Obstetrics* 2014.

6. Davies NE, Matthews LT, Crankshaw TL, Cooper D, Schwartz SR: **Supporting HIV prevention and reproductive goals in an HIV-endemic setting: taking safer conception services from policy to practice in South Africa**. *Journal of the International AIDS Society* 2017, **20**(Suppl 1):21271.

7. Black V, Davies N, Williams BG, Rees HV, Schwartz SR: **Establishing conception intentions and safer conception services for eliminating the vertical, and reducing the horizontal, transmission of HIV**. *BJOG : an international journal of obstetrics and gynaecology* 2016, **123**(10):1585-1588.

8. Mantell JE, Smit JA, Stein ZA: **The right to choose parenthood among HIV-infected women and men**. *J Public Health Policy* 2009, **30**(4):367-378.

9. Schwartz SR, Mehta SH, Taha TE, Rees HV, Venter F, Black V: **High pregnancy intentions and missed opportunities for patient-provider communication about fertility in a South African cohort of HIV-positive women on antiretroviral therapy**. *AIDS and behavior* 2012, **16**(1):69-78.

10. Hancuch K, Baeten J, Ngure K, Celum C, Mugo N, Tindimwebwa E, Heffron R, Partners Demonstration Project T: **Safer conception among HIV-1 serodiscordant couples in East Africa: understanding knowledge, attitudes, and experiences**. *AIDS care* 2018:1-9.

11. Wagner GJ, Woldetsadik MA, Beyeza-Kashesya J, Goggin K, Mindry D, Finocchario-Kessler S, Khanakwa S, Wanyenze RK: **Multi-level Correlates of Safer Conception Methods Awareness and Attitudes Among Ugandan HIV Clients with Fertility Intentions**. *African journal of reproductive health* 2016, **20**(1):40-51.

12. Schwartz SR, Rees H, Mehta S, Venter WD, Taha TE, Black V: **High incidence of unplanned pregnancy after antiretroviral therapy initiation: findings from a prospective cohort study in South Africa**. *PloS one* 2012, **7**(4):e36039.

13. Steiner RJ, Black V, Rees H, Schwartz SR: **Low Receipt and Uptake of Safer Conception Messages in Routine HIV Care: Findings From a Prospective Cohort of Women Living With HIV in South Africa**. *J Acquir Immune Defic Syndr* 2016, **72**(1):105-113.

14. Matthews LT, Moore L, Crankshaw TL, Milford C, Mosery FN, Greener R, Psaros C, Safren SA, Bangsberg DR, Smit JA: **South Africans with recent pregnancy rarely know partner's HIV serostatus: implications for serodiscordant couples interventions**. *BMC public health* 2014, **14**:843.

15. Cohan D, Weber S, Goldschmidt R: **Safer conception options for HIV-serodiscordant couples**. *American journal of obstetrics and gynecology* 2012, **206**(1):e21; author reply e21-22.

16. Chadwick RJ, Mantell JE, Moodley J, Harries J, Zweigenthal V, Cooper D: **Safer conception interventions for HIV-affected couples: implications for resource-constrained settings**. *Topics in antiviral medicine* 2011, **19**(4):148-155.

17. International AIDS Society: **Differentiated Care for HIV: A Decision Framework for Antiretroviral Therapy Delivery**. Durban, South Africa; 2016.

18. **Key considerations for differentiated antiretroviral therapy delivery for specific populations: children, adolescents, pregnant and breastfeeding women and key populations**. Geneva; 2017.

19. Schwartz SR, Bassett J, Sanne I, Phofa R, Yende N, Van Rie A: **Implementation of a safer conception service for HIV-affected couples in South Africa**. *AIDS* 2014, **28 Suppl 3**:S277-285.

20. Schwartz SR, Bassett J, Holmes CB, Yende N, Phofa R, Sanne I, Van Rie A: **Client uptake of safer conception strategies: implementation outcomes from the Sakh'umndeni Safer Conception Clinic in South Africa**. *Journal of the International AIDS Society* 2017, **20**(Suppl 1):21291.

21. Davies N, Mullick S, Schwartz S: **Uptake and clinical outcomes from a primary healthcare based safer conception service in Johannesburg, South Africa: findings at 7 months**.*International AIDS Conference.* Durban, South Africa; 2016.

22. Harris PA, Taylor R, Thielke R, Payne J, Gonzalez N, Conde JG: **Research electronic data capture (REDCap)--a metadata-driven methodology and workflow process for providing translational research informatics support**. *Journal of biomedical informatics* 2009, **42**(2):377-381.

23. Voutilainen A, Pitkaaho T, Kvist T, Vehvilainen-Julkunen K: **How to ask about patient satisfaction? The visual analogue scale is less vulnerable to confounding factors and ceiling effect than a symmetric Likert scale**. *Journal of advanced nursing* 2016, **72**(4):946-957.

24. Torrance GW, Feeny D, Furlong W: **Visual analog scales: do they have a role in the measurement of preferences for health states?** *Med Decis Making* 2001, **21**(4):329-334.

25. Grimsrud A, Barnabas RV, Ehrenkranz P, Ford N: **Evidence for scale up: the differentiated care research agenda**. *Journal of the International AIDS Society* 2017, **20**(Suppl 4):22024.

26. Waldrop G, Doherty M, Vitoria M, Ford N: **Stable patients and patients with advanced disease: consensus definitions to support sustained scale up of antiretroviral therapy**. *Tropical medicine & international health : TM & IH* 2016, **21**(9):1124-1130.

27. Mutasa-Apollo T, Ford N, Wiens M, Socias ME, Negussie E, Wu P, Popoff E, Park J, Mills EJ, Kanters S: **Effect of frequency of clinic visits and medication pick-up on antiretroviral treatment outcomes: a systematic literature review and meta-analysis**. *Journal of the International AIDS Society* 2017, **20**(Suppl 4):21647.

28. Reidy WJ, Rabkin M, Syowai M, Schaaf A, El-Sadr WM: **Patient-level and program-level monitoring and evaluation of differentiated service delivery for HIV: a pragmatic and parsimonious approach is needed**. *AIDS* 2018, **32**(3):399-401.

29. Mindry DL, Milford C, Greener L, Greener RM, Maharaj P, Letsoalo T, Munthree C, Crankshaw TL, Smit JA: **Client and provider knowledge and views on safer conception for people living with HIV (PLHIV)**. *Sexual & reproductive healthcare : official journal of the Swedish Association of Midwives* 2016, **10**:35-40.

30. Finocchario-Kessler S, Wanyenze R, Mindry D, Beyeza-Kashesya J, Goggin K, Nabiryo C, Wagner G: **"I may not say we really have a method, it is gambling work": knowledge and acceptability of safer conception methods among providers and HIV clients in Uganda**. *Health care for women international* 2014, **35**(7-9):896-917.

31. Brown J, Njoroge B, Akama E, Breitnauer B, Leddy A, Darbes L, Omondi R, Mmeje O: **A Novel Safer Conception Counseling Toolkit for the Prevention of HIV: A Mixed-Methods Evaluation in Kisumu, Kenya**. *AIDS education and prevention : official publication of the International Society for AIDS Education* 2016, **28**(6):524-538.

32. Hoffman RM, Jaycocks A, Vardavas R, Wagner G, Lake JE, Mindry D, Currier JS, Landovitz RJ: **Benefits of PrEP as an Adjunctive Method of HIV Prevention During Attempted Conception Between HIV-uninfected Women and HIV-infected Male Partners**. *The Journal of infectious diseases* 2015.

33. The Lancet H: **U=U taking off in 2017**. *The lancet HIV* 2017, **4**(11):e475.

Tableau 1 : Caractéristiques des bénéficiaires des services de conception plus sûre et des participants à l'enquête sur l'évaluation des processus

Caractéristiques	Participants de la cohorte globale (n=692)	Participants à l'enquête sur l'évaluation des processus (n=120)
**Âge, médian (EI)**	34 [30-37]	33 [31-37]
**Sexe, n (%)**		
Féminin	454 (66 %)	78 (65 %)
Masculin	238 (34 %)	42 (35 %)
**Nationalité, n (%)**		
Sud-africaine	379 (55 %)	67 (56 %)
Autre	313 (45 %)	53 (44 %)
**Situation professionnelle, n (%)†**		
En activité	490 (71 %)	85 (71 %)
Sans emploi	200 (29 %)	35 (29 %)
**Niveau d'études, n (%)**		
Niveau primaire ou moins	67 (10 %)	13 (11 %)
3e à terminale	236 (34 %)	38 (32 %)
Élèves inscrits dans un établissement secondaire	318 (46 %)	61 (51 %)
Études supérieures	66 (10 %)	8 (6 %)
**Revenu, médian, en rands (ZAR) [EI]‡**	3300 [1500-5000]	3300 [1600-5150]
**Séropositivité pour le VIH, n (%)**		
Séropositif pour le VIH	591 (86 %)	109 (91 %)
Séronégatif pour le VIH	99 (14 %)	11 (9 %)
**En cas de séropositivité pour le VIH, sous TAR à la période de référence, n (%)**		
Oui	532 (90 %)	100 (92 %)
Non	60 (10 %)	9 (8 %)
**Statut sérologique dans le couple**		
Couple sérodifférent	143 (30 %)	39 (33 %)
VIH + même statut sérologique	219 (46 %)	69 (57 %)
VIH + participant dont le partenaire est de statut sérologique inconnu	117 (24 %)	12 (10 %)

† Absence de données pour n=2. ‡ Absence de données pour n=19.
