# Supplementary material for: Clients’ experiences utilizing a safer conception service for HIV affected individuals: implications for differentiated care service delivery models
Source: Reprod Health. 2019 May 29;16(Suppl 1):65. doi: 10.1186/s12978-019-0718-5 (PMC6538560; doi:10.1186/s12978-019-0718-5)
Supplement: Supplementary file 2 — Translation of the abstract of this article into Portuguese (PDF 120 kb) [file 12978_2019_718_MOESM2_ESM.pdf]

Reproductive Health 2019, 16 (Suppl 1) [translated from the original publication]

Experiências dos clientes que utilizam um serviço de conceção mais seguro para pessoas infetadas com o VIH: Implicações para os modelos de prestação de serviços de cuidados diferenciados

Sheree Schwartz[1,2*], Natasha Davies[2], Nicolette Naidoo[2], Diantha Pillay[2], Nokuthula Makhoba[2], Saiqa Mullick[2]

[1] Department of Epidemiology, Johns Hopkins School of Public Health, Baltimore, MD, USA
[2] Wits Reproductive Health Institute, University of the Witwatersrand, Johannesburg, South Africa

***Autor correspondente:**
Dr. Sheree Schwartz, 615 N. Wolfe St, E7644, Baltimore, MD, USA, 21205. E-mail: sschwartz@jhu.edu

Endereços de e-mail:
Natasha Davies: davies@anovahealth.co.za
Nicolette Naidoo: NNaidoo@wrhi.ac.za
Diantha Pillay: DPillay@wrhi.ac.za
Nokuthula Makhoba: NMakhoba@wrhi.ac.za
Saiqa Mullick: SMullick@wrhi.ac.za

RESUMO

Introdução: Os serviços de conceção mais seguros promovem a saúde e os direitos reprodutivos das famílias, ao mesmo tempo que minimizam os riscos de transmissão do VIH entre casais que estão a tentar conceber, bem como os riscos de transmissão vertical. Os dados de implementação, incluindo as experiências dos clientes que utilizam serviços de conceção mais seguros na África Subsariana, são limitados.

Métodos: O centro comunitário de saúde de Hillbrow começou a oferecer serviços de conceção mais seguros para pessoas e casais infetados com VIH em Joanesburgo, África do Sul, em junho de 2015. Foram recrutados consecutivamente clientes a utilizar serviços de conceção mais seguros para uma subamostra estratificada, desde abril de 2016 até agosto de 2017, para uma entrevista transversal que avaliou as perceções dos clientes em relação à aceitabilidade e ao valor dos serviços, bem como o conhecimento e a eficácia pessoal em termos de conceção mais

segura. Também foi utilizada uma escala analógica visual de 0 a 100 para avaliar as experiências dos clientes, cujas pontuações foram classificadas em termos de aceitação, valor, conhecimento e eficácia pessoal como reduzida (<50), moderada (entre ≥50 e ≤79) e elevada (≥80), respetivamente. A comparação das pontuações foi efetuada após presença nas consultas de conceção mais segura.

Resultados: Entre os 692 clientes a utilizar serviços de conceção mais seguros, foram incluídos na amostra 120 (17%) para o processo de avaliação, sendo as caraterísticas da subamostra dos participantes semelhantes ao grupo global. Os clientes atribuíram uma pontuação média de ≥90 para cada questão que avaliava a aceitabilidade do serviço, com 96% (114/119) a indicar um valor de perceção elevada (pontuações ≥80) para consultas regulares de conceção mais segura até ao momento de conceção. 58% dos clientes (n=70) indicaram que adquiriram novos conhecimentos durante a consulta concluída no dia do inquérito, apesar de a aquisição de novas informações apresentar uma tendência para diminuir com a continuação das consultas (p=0,09). Em termos de estratégias de conceção mais seguras, 80% dos clientes afirmaram ter um elevado nível de conhecimento sobre o impacto do tratamento antirretroviral (TAR) e supressão vírica da transmissão do VIH, 67% indicaram um nível elevado de conhecimento sobre a importância do rastreio de IST e 56% relativamente à limitação de sexo sem preservativo nos dias de fertilidade máxima; 34% de casais serodiferentes afirmaram ter um conhecimento elevado sobre profilaxia de pré-exposição. O nível de eficácia pessoal variou entre métodos de conceção mais seguros e foi semelhante em todas as consultas do estudo.

Conclusões: Os clientes consideraram as consultas de conceção mais segura valiosas e preferiram ter uma presença regular nas mesmas até ao momento de conceção, contudo foi

possível observar uma estagnação nos conhecimentos e eficácia pessoal durante as consultas subsequentes após a participação inicial nos cuidados de conceção mais segura. Os serviços mais intensivos poderão ser mais adequados para determinados clientes com base em circunstâncias clínicas, mas muitos casais podem potencialmente receber uma abordagem "mais leve" e minimizar na mesma os riscos de transmissão do VIH.

Palavras-chave: Conceção mais segura, VIH, prevenção do VIH, cuidados diferenciados, casais discordantes, implementação

Sobre este suplemento

Este resumo foi publicado como parte da revista científica *Reproductive Health*, Volume 16, Suplemento 1, 2019: Integração Eficaz dos Serviços de Saúde Sexual e Reprodutiva e de Prevenção, Cuidados e Tratamento do VIH na África Subsariana: Onde estão as provas da implementação do programa?
O suplemento foi publicado como uma colaboração entre as revistas científicas *Reproductive Health* e *BMC Public Health.* O conteúdo integral, incluindo as versões em francês, português e inglês, estão disponíveis online:
https://bmcpublichealth.biomedcentral.com/articles/supplements/volume-19-supplement-1
e
https://reproductive-health-journal.biomedcentral.com/articles/supplements/volume-16-supplement-1
